# Islands Within Islands: Bacterial Phylogenetic Structure and Consortia in Hawaiian Lava Caves and Fumaroles

**DOI:** 10.3389/fmicb.2022.934708

**Published:** 2022-07-21

**Authors:** Rebecca D. Prescott, Tatyana Zamkovaya, Stuart P. Donachie, Diana E. Northup, Joseph J. Medley, Natalia Monsalve, Jimmy H. Saw, Alan W. Decho, Patrick S. G. Chain, Penelope J. Boston

**Affiliations:** ^1^Department of Environmental Health Sciences, Arnold School of Public Health, University of South Carolina, Columbia, SC, United States; ^2^School of Life Sciences, University of Hawai‘i at Mānoa, Honolulu, HI, United States; ^3^Department of Microbiology and Cell Science, University of Florida, Gainesville, FL, United States; ^4^Department of Biology, University of New Mexico, Albuquerque, NM, United States; ^5^Department of Biological Sciences, The George Washington University, Washington, DC, United States; ^6^Biosciences Division, Los Alamos National Laboratory, Los Alamos, NM, United States; ^7^National Aeronautics and Space Administration (NASA) Ames Research Center, Moffett Field, CA, United States

**Keywords:** lava caves, fumaroles, taxonomic distinctness, networks, cave microbiology, microbial consortia, volcanic environments

## Abstract

Lava caves, tubes, and fumaroles in Hawai‘i present a range of volcanic, oligotrophic environments from different lava flows and host unexpectedly high levels of bacterial diversity. These features provide an opportunity to study the ecological drivers that structure bacterial community diversity and assemblies in volcanic ecosystems and compare the older, more stable environments of lava tubes, to the more variable and extreme conditions of younger, geothermally active caves and fumaroles. Using 16S rRNA amplicon-based sequencing methods, we investigated the phylogenetic distinctness and diversity and identified microbial interactions and consortia through co-occurrence networks in 70 samples from lava tubes, geothermal lava caves, and fumaroles on the island of Hawai‘i. Our data illustrate that lava caves and geothermal sites harbor unique microbial communities, with very little overlap between caves or sites. We also found that older lava tubes (500–800 yrs old) hosted greater phylogenetic diversity (Faith's PD) than sites that were either geothermally active or younger (<400 yrs old). Geothermally active sites had a greater number of interactions and complexity than lava tubes. Average phylogenetic distinctness, a measure of the phylogenetic relatedness of a community, was higher than would be expected if communities were structured at random. This suggests that bacterial communities of Hawaiian volcanic environments are phylogenetically over-dispersed and that competitive exclusion is the main driver in structuring these communities. This was supported by network analyses that found that taxa (Class level) co-occurred with more distantly related organisms than close relatives, particularly in geothermal sites. Network “hubs” (taxa of potentially higher ecological importance) were not the most abundant taxa in either geothermal sites or lava tubes and were identified as unknown families or genera of the phyla, Chloroflexi and Acidobacteria. These results highlight the need for further study on the ecological role of microbes in caves through targeted culturing methods, metagenomics, and long-read sequence technologies.

## Introduction

Volcanic features in Hawai‘i, such as lava caves and fumaroles, are often oligotrophic, yet they host unexpectedly high microbial diversity (Northup and Lavoie, 2015; Riquelme et al., 2015; Wall et al., 2015). Lava caves and tubes form when the surface of flowing molten lava cools, crust over, and the molten lava beneath continues to flow, leaving tube-like structures or pockets behind that to form caves. Temperature and humidity within caves vary less than over the surface terrain above, and sunlight penetrates over cave and tube entrances, or through “skylights” where the cave ceiling collapses. In such environments, several studies have documented high microbial diversity from around the world, including Iceland, the Azores, the Canary Islands, and Hawai‘i (Gabriel and Northup, 2013; Northup and Lavoie, 2015) with high levels of endemism (Snider, 2010).

Fumaroles and geothermally active lava caves are more dynamic, extreme environments that are formed when steam and volcanic gases exit cracks in basalt deposits, creating vents (Ellis et al., 2008; Costello et al., 2009; Wall et al., 2015). Fumaroles are far less studied than lava caves, even though they are one of the most common geothermal features on Earth (Bizzoco and Kelley, 2019). Wall et al. (2015) studied samples from deposits in contact with steam in fumaroles on the island of Hawai‘i and concluded that Hawaiian fumaroles are biodiversity hotspots, with exceptionally high diversity. Lava caves above deeper situated magma can also have characteristics similar to fumaroles, with higher temperatures, deposits on cave walls that arise through pH reductions caused by volcanic gasses, and high humidity from magmatic heating of air and groundwater. These geothermally active sites represent a unique type, or early stage in the development of volcanic ecosystems, often occurring on younger basalts that are less oxidized and weathered. They also often harbor a wide variety of chemolithotrophic microbes and other extremophiles (Wall et al., 2015). Therefore, lava tubes and geothermal sites present a spectrum of environmental conditions and community stability in volcanic ecosystems and provide excellent systems which help identify and study drivers of bacterial community diversity and structure.

Volcanic environments are also systems in which the formation and persistence of robust and adaptable microbial consortia may be critical for community survival in extreme conditions, including those characterized by low nutrient availabilities or extreme temperatures. Microbial consortia are defined here as consistent subgroups of bacteria in a community that occur and function together, and which may possess emerging, synergistic activities that cannot be attributed to any one species in the group (Bosse et al., 2015; Vishwakarma et al., 2020). In volcanic ecosystems, microbial neighbors may be highly interdependent, particularly in relationships with chemolithotrophs which can utilize reduced compounds in basalts as energy sources (Gomez-Alvarez et al., 2007; Northup and Lavoie, 2015). These may be “hub” organisms, the community members that have ecologically important roles and which can be identified through network analysis (Toju et al., 2018; Zamkovaya et al., 2020). Hub organisms might also include bacteria that are capable of photoautotrophy in very low light levels, such as cave-dwelling Cyanobacteria like *Gloeobacter kilaueensis* (Saw et al., 2013).

Previous studies have concluded that subsurface microbial consortia establish small-scale, site-specific microbial–mineral interactions over time, such as when bacteria acclimate and adapt to specific mineral chemistry, nutrient, and atmospheric conditions over microscopic scales (Miller et al., 2014; Barton, 2015). Experimental studies of biofilm-mineral interactions related to caves or basalts have also been completed, but our understanding of these interactions remains limited (Edwards et al., 2005; Cockell et al., 2020). Jones and Bennett (2014) found that in experiments using pure and mixed cultures of *Thiothrix unzii*, the competitive exclusion between species, as well as other factors, were related to biomass density and diversity on mineral and rock surfaces. This would create a pattern of high diversity at very small spatial scales that are dependent on the type of minerals in the basalt and other micro-scale environmental variables that are difficult to observe and record in the field.

Large-scale ecological factors may also influence bacterial diversity in lava caves and fumaroles, including the age and degree of basalt weathering, and stochastic and deterministic processes that shape microbial communities over time. For example, in a study of Hawaiian volcanic deposits, increasing deposit age was generally accompanied by increasing concentrations of organic matter and microbial biomass (King, 2007). Northup and Lavoie (2015) also noted that lava caves provide an opportunity to study microbial successional patterns as microbes invade fresh lava tubes and diversify over time, with additional microbial introductions through water and sediments that enter the caves, animal entry, aerosols, and penetrating plant roots. However, such patterns and their potential underlying drivers, including mineral composition-driven selection (i.e., habitat filtering by the environment) or competitive exclusion due to low nutrient availability have not been investigated in lava tubes or fumaroles. However, stochastic and deterministic patterns that drive community structure can be investigated through the study of the phylogenetic structure or “breadth,” which determines the phylogenetic signature, or taxonomic distinctness, of a community, and how well a given sample represents the larger, regional pool of the given taxa (see Clarke and Warwick's taxonomic distinctness; Clarke and Warwick, 1998, 2001).

In this study, 70 samples were collected from lava tubes and geothermal sites (including fumaroles and geothermal caves) on the island of Hawai‘i between 2006 and 2009 and 2017 and 2019. Data were used to investigate the drivers of phylogenetic distinctness and diversity in these features and their microbial consortia, which may, in turn, contribute to the high bacterial diversity observed in lava caves and fumaroles. We hypothesized that older lava tubes would have higher phylogenetic diversity, likely due to the greater stability of environmental conditions in lava tubes and greater bio-weathering of the rock, thus making nutrients more readily available. We also hypothesized that lava tubes would provide for greater complexity in co-occurrence networks due to the development of unique microbial consortia at very small spatial scales due to the mineralogical variability at those small scales, which could create complex interactions over time.

Using massively parallel sequencing of a fragment of the 16S rRNA gene from microbial communities of lava tubes and geothermal sites, this study investigated the following: (1) the effect of age of the surface lava flow, and other environmental and study variables, on bacterial phylogenetic diversity, (2) if bacterial phylogenetic structure occurs by chance, or if over-dispersion or habitat filtering is evident, and (3) co-occurrence patterns of bacteria through network analysis to determine hubs and phylogenetic diversity within bacterial consortia at small spatial scales. Understanding the phylogenetic distinctness, diversity patterns, and microbial consortia in natural, volcanic environments will address questions in a variety of fields, including the structure of possible microbial communities on an early Earth and Mars in astrobiology studies, potential use of microbial consortia in biotechnology and bioremediation, and improving our understanding of microbial communities in volcanic or desert soils for enhanced agricultural production.

## Materials and Methods

### Sampling Sites

In total, 70 samples were collected over multiple years from lava caves and fumaroles across the island of Hawai‘i. Samples collected from Kaūmana cave did not require a permit because they were microbiological in nature, and we did not collect rock or damage surfaces. All other samples, except for geothermal caves within Kīlauea Caldera (see below), were collected from caves or vents on private lands, and permission was granted for the collection. Samples varied in terms of date of collection, type of material, DNA extraction method, estimated age of the basalt, the temperature at the time of collection, elevation, and average annual rainfall received at the surface (Supplementary Table 1). Lava flow age provides an approximate age of a cave or fumarole, and for each site, the approximate age was determined from a US Geological Survey (USGS) National Geologic database (https://ngmdb.usgs.gov/ngmdb/ngmdb_home.html) and in communication with the Hawaiian Volcano Observatory (HVO). Annual rainfall amounts were collected from the Rainfall Atlas of Hawai‘i (http://rainfall.geography.hawaii.edu; Frazier et al., 2016). Estimates of the elevation were determined during sample collection or from Google Earth using GPS coordinates of each site.

Lava caves in the 1922 lava flow in Kīlauea Caldera, Hawai‘i, were sampled between 2006 and 2009 under permit #HAVO-2009-SCI-0029 issued to Stuart P. Donachie by the Hawai‘i Volcanoes National Park (National Parks Service, Department of the Interior) and were the only samples collected in this study that required a permit. Based on the time of the last lava flow, the caves are thought to be ~100 years old. Cores of epilithic biofilms were collected from each cave wall directly into sterile cryovials. Conditions differed among the caves, with temperatures ranging from ambient at the surface to almost 46°C, and relative humidity from ambient to 102%. Cave floor temperatures ranged from 20 to over 60°C. For example, in Big Ell cave, the mean air temperature recorded by a Hobo data-logger (Bourne, Massachusetts) for the 4 months in the summer of 2006 was 26°C, with a range from 17.9 to 45.9°C (data not shown). Water in the cave mostly occurred in the form of dripping condensation that exceeded meteoric rainfall on the surface by about six times. Heated groundwater in Big Ell drove relative humidity in the same period to a mean of 95.2% (range, 52.25–102.25%).

Samples were collected from Pahoa and Kaumana caves in 2017; both are lava tubes on the windward side of the island of Hawai‘i. Pahoa cave is located in flows that dated to 300–750 years of age, and the cave is considered to be older than 500 years (pers. comm., HVO, USGS). Kaumana cave is much younger, at ~130 years, and formed in lava flows from Mauna Loa in 1881. Samples from Pahoa and Kaumana were collected by scraping a 1 cm^2^ area of the cave wall with a sterile spatula into a cryovial. These samples were stored on ice until arrival the same day at the University of Hawai‘i at Mānoa, whereupon they were transferred to a −80°C freezer until DNA was extracted, as described below.

Samples were collected from the Kipuka Kanohina Cave System on the southeast side of the island of Hawai‘i in December 2017, January 2018, and January 2019, with the permission of the landowners. Sites sampled included the Kula Kai Caverns, Tapa, and Maelstrom segments, all of which were ~800 years old. Here, small pieces of rock with epilithic biofilms, spheroids, coralloids, moonmilk, or mineral crusts were chipped from the cave wall with a flame-sterilized chisel. A sterile scopula also scraped ooze from cave walls. Separate samples were placed directly into sterile 50 ml polypropylene tubes and immediately covered with a sterile sucrose lysis buffer and sealed with parafilm after recapping. Samples were stored in a refrigerator until transported to the Northup lab at the University of New Mexico, where they were stored in a −80°C freezer until DNA extraction, as described below.

Samples were taken from several centimeter thick biofilms in the vicinity of steam vents in the East Rift Zone located near the town of Pahoa, Hawai‘i, in August 2019. These fumaroles occur on two lava flows, with one dating to ~65 years ago and another to ~400 years ago (pers. comm, HVO, USGS). Therefore, these samples were assigned a wide age range (64–400 years). Samples were collected directly into sterile 15 or 50 ml polypropylene tubes, which were sealed with parafilm immediately after recapping, and stored on ice during transport the same day to a nearby −20°C freezer. Frozen samples were wrapped with ThermoSafe U-tek cold packs at −23°C and shipped to the University of Hawai‘i at Mānoa or the George Washington University. Samples were then immediately placed in a −80°C freezer until DNA extraction.

### DNA Extraction

The DNA extraction method varied depending on the sample type (Supplementary Table 1). Extractions of DNA from samples taken from the Pahoa, Kaumana, Big Ell, Big Mouth, Ahu Too caves, and some steam vent samples were extracted using the Qiagen DNeasy PowerSoil DNA Kit (Germantown, MD) according to the instructions of the manufacturer and included a bead-beating step of 1.5 min in a Mini-Beadbeater-24 (BioSpec Products, Inc., Bartlesville, OK). Samples from the Kipuka Kanohina cave system were also extracted with the Qiagen DNeasy PowerSoil DNA Kit with a BioSpec Products Mini-Beadbeater-8 for 1.5 min at medium intensity and 50 μl of powdered skimmed milk (Difco) incorporated into the extraction protocol before bead-beating in order to increase the DNA yield (Takada-Hoshino and Matsumoto, 2004). DNA extracted according to these methods was shipped to Los Alamos National Laboratory (LANL) for 16S rRNA amplicon sequencing on an Illumina MiSeq.

DNA from samples collected along the East Rift Zone steam vents (referred to here as Pahoa Steam Vents) was extracted using the ZymoBIOMICS DNA Kit according to the manufacturer's instructions (Zymo Research, Orange CA, USA). The extracted DNA was sequenced at the Advanced Studies in Genomics, Proteomics and Bioinformatics (ASGPB) core DNA sequencing facility at the University of Hawai‘i at Mānoa using the Illumina MiSeq platform. Both LANL and ASGPB sequencing facilities used the same protocols, kits, and primers for sequencing (refer to the section below).

### 16S rRNA Gene Amplicon Sequencing

Degenerate primers 341F (5′CCTACGGGAGGCAGCAG 3′) and 806R (GGACTACHVHHHTWTCTAAT-3′) were used to amplify the V3–V4 region of 16S rRNA genes in all samples. The first round of PCR amplified the V4 region from 10 ng of DNA using the KAPA HiFi HotStart Ready Mix and denaturation at 95°C for 3 min. This was followed by 20 cycles of 95°C for 30 s, 55°C for 30 s, 72°C for 30 s, an extension step of 72°C for 5 min, and a final hold at 4°C. The second round of PCR used Nextera XT v2 indices (Illumina Inc., San Diego, CA, USA), with denaturation at 95°C for 3 min, 8 cycles of 95°C for 30 s, 55°C for 30 s, and 72°C for 30 s, followed by an extension of 72°C for 5 min, and a final hod at 4°C. Amplicons were cleaned using AMPure XP beads (Beckman Coulter Inc., Brea, CA, USA).

Unique barcodes allowed multiple amplicons to be pooled for sequencing. The concentration of the pooled amplicons was determined in the Qubit dsDNA HS Assay (ThermoFisher Scientific, Waltham, MA, USA). The average size of the library was determined by the Agilent High Sensitivity DNA Kit (Agilent Technologies, Inc., Santa Clara, CA, USA). Accurate library quantification was determined with the Library Quantification Kit, Illumina/Universal Kit (KAPA Biosystems, Cape Town, South Africa). The amplicon pool was sequenced on an Illumina MiSeq at either Los Alamos National Laboratory (LANL) or in the ASGPB core facility at the University of Hawai‘i at Manoa. Both sequencing facilities generated paired-end 301 bp reads, with the expectation that each sample would provide 50,000–100,000 reads. Sequences were submitted to Dyrad and are publicly available at https://doi.org/10.5061/dryad.z612jm6f0.

### Analyses

Raw sequence data were imported to the EDGE Bioinformatics platform (Li et al., 2017), utilizing QIIME 2 (vQIIME 2.4.1; Bolyen et al., 2019). Raw sequences were demultiplexed and denoised using the DADA2 pipeline (Callahan et al., 2016; command qiime dada2 denoise-paired). The 5′ ends of both forward and reverse sequences were trimmed by 20 base pairs (bp) to remove PCR primers. The 3′ end of the reverse read was truncated at 200 bp. Representative sequences for each amplicon sequence variant (ASV) were used for taxonomic classification (command q2-feature-classifier; Bokulich et al., 2018). A phylogenetic tree was generated using MAFFT alignment (Katoh et al., 2002) and FastTree (Price et al., 2009), using the command qiime phylogeny align-to-tree-mafft-fasttree, followed by alpha and beta diversity analyses (command qiime diversity core-metrics-phylogenetic). Taxonomic classification of ASVs was completed using an in-house subset of the SILVA database (v132; Yilmaz et al., 2014) for the V3–V4 region only, based on the primer set 341F and 806R. The data were then split into two sets for most analyses: lava tubes (*n* = 38), and geothermal sites (*n* = 32), the latter of which included geothermally active caves and fumaroles. Bar graphs of community composition at the class level (L3) were created in numbers (version 11.2 7032.0.145), with the proportion of each class-level taxon in each site calculated from raw abundance tables generated in EDGE-QIIME2.

### Multivariate Analyses

For multivariate statistical analyses and exploration of data, abundance tables and distance matrices generated in the EDGE-QIIME2 platform were imported into PRIMER-e (v7.0). Principal Coordinates Analysis (PCoA) was completed using distance matrices of weighted Unifrac distances (Lozupone et al., 2005). All 70 samples were run together, based on rarefied data (minimum # sequences/per sample = 5,471), with the exploration of differences in DNA extraction methods, evaluated sequencing, and environmental variables (Supplementary Table 1). PCoA plots based on rarefied data were also generated from the two individual datasets of lava tubes (*n* = 38; minimum number of sequences/per sample = 5,471) and geothermal sites (*n* = 32; minimum number of sequences/per sample = 14,364).

### Community Structure and Phylogenetic Distinctness Analysis

Average taxonomic distinctness (Delta+; referred to as *phylogenetic* distinctness here because the data are based on a phylogenetic tree) summarizes features of the overall hierarchical structure of an ecological community based on a phylogenetic tree generated from that community's genetic information. This is calculated using a phylogenetic tree of all sequences (16S rRNA amplicon sequences in this study) from all samples and determination of the patristic distances between each pair of ASVs (*i* and *j*). Phylogenetic distinctness equals the average phylogenetic distance between species in a community. The variance of phylogenetic distinctness (Lambda+) equals the variance of the phylogenetic distances {ω_ij_} between each pair of sequences, about their mean value (Delta+). These measurements are not dependent on sampling size or effort, unlike Faith's phylogenetic diversity, which increases with the increasing number of taxa, and therefore requires the use of rarefied sequencing data.

Average phylogenetic distinctness also provides a framework that tests departures from expectations through permutation tests, completed here through TAXDTEST in PRIMER-e (v7.0). A sample randomly drawn from the entire list of organisms in the tree can be taken to test if the observed subset of organisms in an actual sample represents the phylogenetic biodiversity expressed in the full phylogenetic inventory. Such information could help support or reject hypotheses about microbial interactions and the importance of syntropy in microbial consortia utilization of nutrients in the rock, and of other limited resources in caves.

To determine the average and variance of phylogenetic distinctness, a pairwise distance matrix of the rooted phylogenetic tree of all representative ASVs detected across all 70 samples (9,965 ASVs) was calculated in Geneious Prime (v2021.2.1), using the patristic distance calculation. Patristic distance is equivalent to cophenetic distance and is defined as the sum of the branch lengths between two pairs of ASVs (tips of the branches). The branch length is given in average nucleotide substitutions and is calculated back to their most recent common ancestor.

The patristic distance matrix generated in Geneious was then imported into PRIMER-e for calculations of average phylogenetic distinctness per sample (avgTD or Delta+) and variation around the mean of phylogenetic distinctness (varTD or lambda+; Clarke and Warwick, 1998, 1999, 2001). Delta+ is a univariate biodiversity index, which in this case, calculates the average patristic distance between all pairs of ASVs in a sample, and the distance is defined as the path length through a phylogenetic tree connecting these species. Using permutation tests, we then compared the actual Delta+ and Lambda+ of each sample to the null model of the phylogenetic community structure of each sample, which was random (i.e., no evidence of habitat filtering or over-dispersion). TAXDTEST routine completes permutation tests by selecting random subsets of ASVs from the phylogenetic tree distance matrix at various sample sizes, thereby testing for a departure from the null model of all ASVs in the phylogeny which has an equal probability of being included in any random sample draw (without replacement) and evaluates the phylogenetic relatedness and breadth of the community (Clarke and Warwick, 1998). This process generated a 95% probability funnel that illustrates the Delta+ and Lambda+ across the range of ASVs of our samples (**Figure 5**) and illustrates whether ASVs in a sample present a higher or lower than expected phylogenetic relatedness. If a sample falls near the mean predicted Delta+ or Lambda+ inside the 95% probability funnel, the null hypothesis cannot be rejected, and communities appear to be assembled in a random manner. If it falls either above (over-dispersion) or below (phylogenetic clustering) the mean, the null model is rejected, and communities do not appear to be assembled in a random manner.

### Network Construction

To evaluate bacterial co-occurrence patterns in microbial communities from the geothermal sites and lava tubes sampled here, including the overall connectivity and structure of the dominant species of these systems, network models and metrics were evaluated (refer to Zamkovaya et al., 2020 for detailed methods). By mathematically modeling a microbial community as a network, wherein nodes are different species and edges represent their relationships (Wuchty et al., 2006; Ma'ayan, 2011), we can depict species interactions and study the interactions among and structure of the community and environment. Network metrics, such as hub score, betweenness centrality, closeness centrality, and degree centrality (Huang, 2004; Blüthgen et al., 2008; Ma'ayan, 2011), can be used to quantitatively assess these communities and may help identify important taxa in each environment, thereby providing important clues about how specific taxa or gene products may contribute to the function of an ecosystem (Gehlenborg et al., 2010).

To generate networks, each abundance table, taxonomy table, and rooted phylogenetic tree generated by EDGE-QIIME2 was imported into R with its corresponding sample metadata information using the QIIME2R (v0.99.35) and phyloseq (v1.30.0) packages and the qzatophyloseq() function. For each environment, using the get_back_res_meeting_min_occ() function from the mdmnets (v0.1.0) package (Zamkovaya et al., 2020), the ASV phyloseq object was first filtered to include taxa present across the majority (15 and 12.5% for lava tubes and geothermal sites, respectively) of samples and then normalized, transformed, and converted into an inverse covariance adjacency matrix, using the Meinshausen–Buhlmann (MB) neighborhood selection (method = “mb” parameter) algorithm [SpiecEASI (v.1.0.7) package; Kurtz et al., 2015]; this estimated the conditional dependence of each pair of ASVs and the stability approach to regularization selection (StARS) variability (i.e., minimum λ) threshold set to default (0.05; Liu et al., 2010), to calculate the most optimal, sparse, and direct co-occurrence relationship among ASVs. Each adjacency matrix was then converted into an igraph object and visualized as a network using the adj2igraph() and plot_network() functions from SpiecEasi. For both lava cave and geothermal sites, networks for all classification levels (phylum, class, family, order, genus) were constructed using the get_net_plots_all_ranks() function, and hub networks for all classification levels were created using the get_hub_plots_all_ranks() function from the mdmnets package. Network attributes (number of nodes, number of edges, and network density) were calculated using the igraph package and theV(), E(), and edge_density() functions.

For each dataset (geothermal vs. lava tubes), subnetworks (sub-communities of the initial igraph object of the adjacency matrix) were extracted and calculated using the Louvain hierarchical multi-level modularity optimization algorithm cluster_louvain() function from the igraph package, which detects community structure in larger networks (Blondel et al., 2008). This scalable, greedy optimization method identifies communities from large networks by identifying modules (i.e., subnetworks) in the larger network through a measure of modularity. This is a scale value ranging from −0.5 to 1, which is based on the relative density of edges (actual number of edges/possible number of edges) inside each module in the larger network compared to the density of edges outside each module. The Louvain method first assigns each node in the larger network to its own local community, and in a greedy, hierarchical approach, reassigns and groups each node to the community to which it contributes the most value in its modularity measure. It then repeats and restarts these calculations until no nodes can be reassigned and end only when there is a single node left, or when the modularity of a community cannot be increased further. The modules identified in the lava tube and geothermal environments were then visualized as subnetworks using the get_net_plots_all_ranks() function and modified R scripts. The community number and overall community size of each node were saved and added to the other list of features (node number, classification, degree centrality score, betweenness centrality score, and hub score).

Using only ASVs that occurred in subnetworks, ASVs were clustered into operational taxonomic units (OTUs) at 98.6% sequence similarity using open-reference OTU picking in QIIME2 to reduce the likelihood of building a phylogenetic tree of subnetwork members that would contain sub-strains. OTU-clustered ASVs were then aligned in QIIME2 using MAFFT (Katoh et al., 2002) and FastTree (Price et al., 2009; command qiime phylogeny align-to-tree-mafft-fasttree) to build a phylogenetic tree. Newick tree (.tre) files generated in QIIME2 were imported into the Interactive Tree of Life v6.4.3 (ITOL: https://itol.embl.de; Letunic and Bork, 2021) to generate tree images. The subnetwork of a given ASV was added to the tree images as metadata in ITOL.

### Shared ASV Comparison

The number of ASVs shared among lava tubes and geothermal samples was calculated and visualized using the UpSetR (v1.4.0) package, and the upset() function on the transformed ASV abundance table. Upset plots are an improved alternative to Venn diagrams, particularly when dealing with more than 3 or 4 groups, that visualize set intersections in a matrix layout. All upset plots were created in R, using ggplot2 (v3.3.2) and UpSetR packages, and with custom color palettes for each environment in the RColorBrewer (v1.1.2) package.

## Results

### Community Composition and Environmental Variables

High-throughput sequencing of 16S rRNA gene fragments from samples of lava caves and geothermal sites on the island of Hawai‘i produced 10,715,694 raw sequences, with a mean length of 300.46 bp (base pairs). After demultiplexing, the minimum number of sequences in each sample was 5,541, and the maximum number was 346,293, with a median sequence count per sample of 26,622. For geothermal sites only (*n* = 32), the minimum number of sequences in a sample was 14,365, with a median sequence count of 176,380, and a maximum of 346,293. A total of 4,296 ASVs was identified in unrarefied data. In the lava tube dataset, the minimum number of sequences in each sample was 5,541, with a maximum of 42,281, and a median of 16,651. The total number of ASVs from the lava tubes was 6,036 (unrarefied).

**Figure 1:**
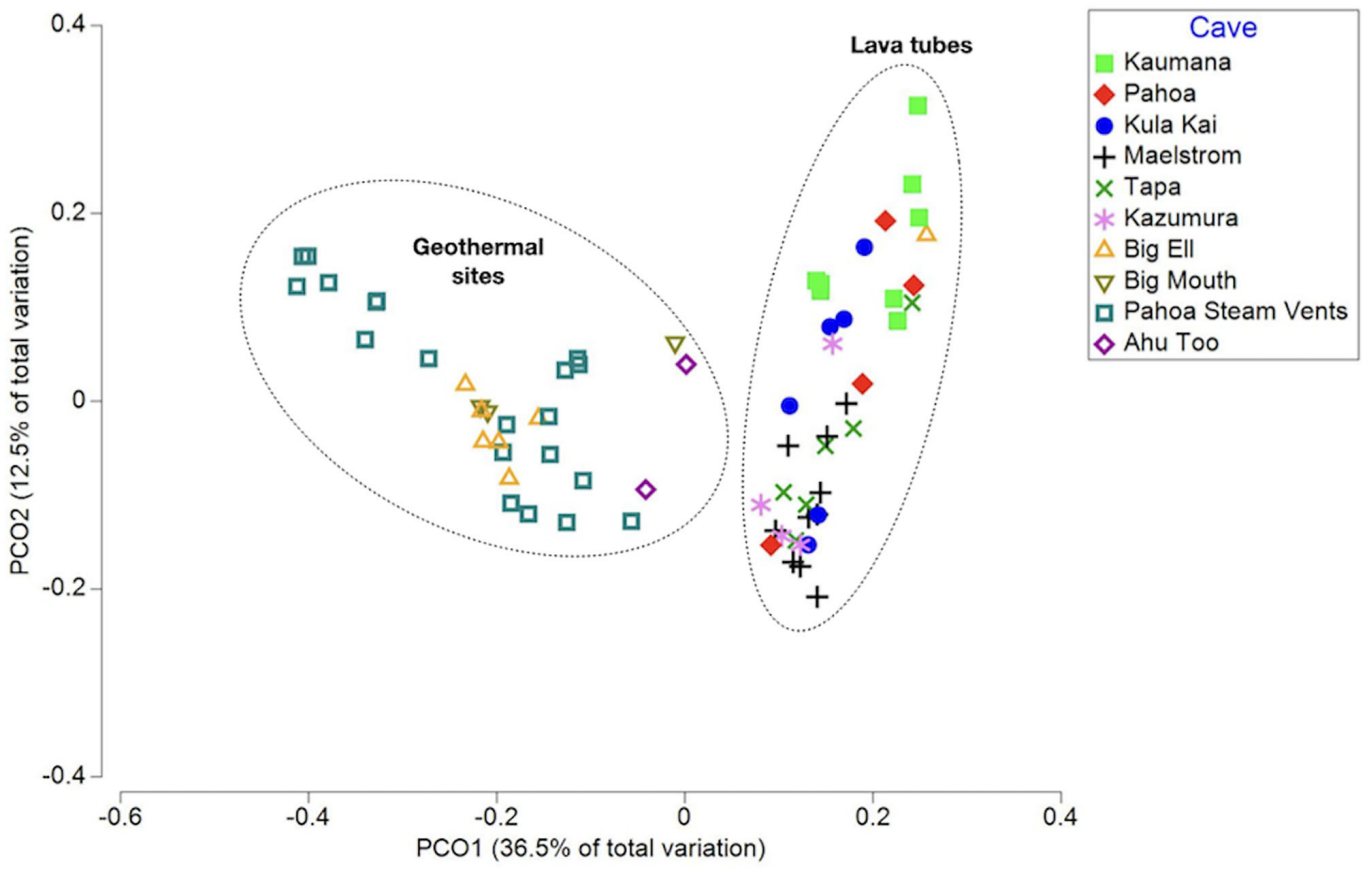
A principal coordinates analysis (PCoA) of the weighted Unifrac distance measured between samples, capturing 49% of the variation in multivariate space. Samples are displayed as the cave or site they were collected from. This illustrates that samples from geothermal sites and lava caves are two distinct populations in terms of phylogenetic community composition and abundance.

Rarefaction curves suggest that the sequencing depth was adequate to capture most of the diversity in most samples (Supplementary Figure 1). A total of 9,965 ASVs (i.e., taxa) were identified in unrarefied data for all 70 samples; these were used in co-occurrence network construction and phylogenetic distinctness analyses. Beta diversity analyses were completed using rarefied ASV tables. Environmental variables explored through multivariate analyses to detect ecological patterns included sample type (i.e., biofilm, ooze, mineral crust, etc.), average annual rainfall at surface, cave/site, temperature, cave age, elevation, year of collection, DNA extraction method, and sequencing lab (Supplementary Table 1). The PCoA of weighted Unifrac distances, a beta-diversity measure that incorporates phylogenetic diversity, abundance, and composition of samples, found that samples did not group consistently with the environmental variable rainfall at the surface, elevation, or cave/site, but instead clustered as geothermal sites and lava tubes (Figure 1). This was further supported by a Kruskal–Wallis analysis of the alpha-diversity measure, a phylogenetic diversity (Faith's PD; Faith, 1992; Kruskal-Wallis: H = 17.3; *p* = 0.0002; pairwise test results in Supplementary Table 2). Geothermal caves, which are the remains of inflation bubbles in lava flows near Kīlauea Caldera, were not statistically different from fumaroles in terms of Faith's PD (Kruska–Wallis pairwise test: H = 0.183; *q* = 0.668; Supplementary Table 2). Based on the outcomes of these analyses, some further analyses were applied separately to lava tubes and all geothermal sites. The PCoA also found no relationship or clustering of samples associated with the “DNA extraction method” or “sequencing facility,” suggesting the methods and facilities used produced comparable results (Supplementary Figures 2, 3). Samples collected from geothermal caves in Kīlauea Caldera were the only sites that were collected twice (in 2006 and 2019), and these samples clustered together as geothermal sites in PCoA analysis, but no other samples clustered together as a factor of date of collection (Supplementary Figure 4).

Additional multivariate analysis of the weighted Unifrac distances using PCoA of all 70 samples and separate analyses of geothermal sites, suggests that the temperature of the cave or site may be the driver of community composition and abundance, with sites above 45°C grouping together. However, this was not further supported by a pairwise Kruskal–Wallis analysis of Faith's PD. Similarly, within lava tubes, microbially-mediated mineral deposits (i.e., mineral crusts, coralloids, etc.) were grouped together in PCoA analysis, but such groupings were not found in comparisons of all 70 samples, and there were no statistically significant differences found among pairwise Kruskal–Wallis tests to support these differences, likely due to the small sizes of various sample types.

**Figure 2:**
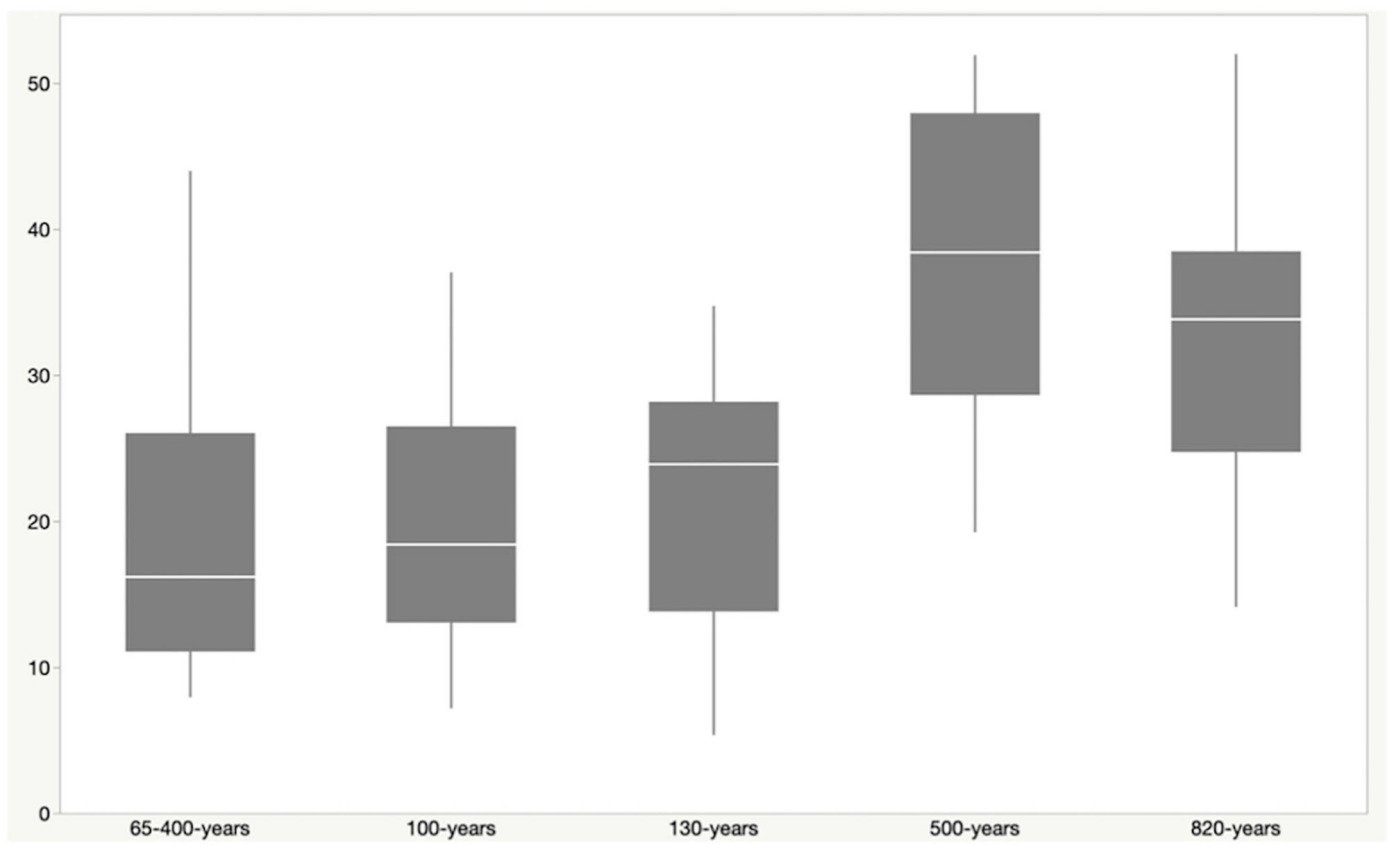
Faith's Phylogenetic Diversity (PD) vs. estimated site age. The approximate age of a particular site was defined by the latest flow recorded for the location. Steam vents (sites P & S) are features in two possible flow events and were thus given the range of 65–400 years, representing the earliest and latest potential time periods for those flows. Phylogenetic diversity increases with the increasing age of the lava flow at that site. PD was higher among sites that were 500–820 year old, when compared to younger sites. This includes Kaumana cave, a lava tube, which is only ~130 years old.

Phylogenetic diversity was higher in sites from older lava flows than younger flows, with site age estimated from the time of the most recent lava flows on the surface (Figure 2). Faith's PD was higher in samples from sites that were between 500 and 820 years old when compared to sites up to 400 years old (Kruskal–Wallis: H = 24.1; *p* = 0.00007; pairwise test results in Supplementary Table 3). Although most of the geologically younger sites are from geothermal locations, Kaumana cave is ~130 years and is a lava tube that is not geothermally active, suggesting that the age of basalt is related to diversity. Kaumana cave hosted less diversity relative to other lava tubes, but the difference was not statistically different (Kruskal–Wallis: H = 8.3; *p* = 0.141).

**Figure 3:**
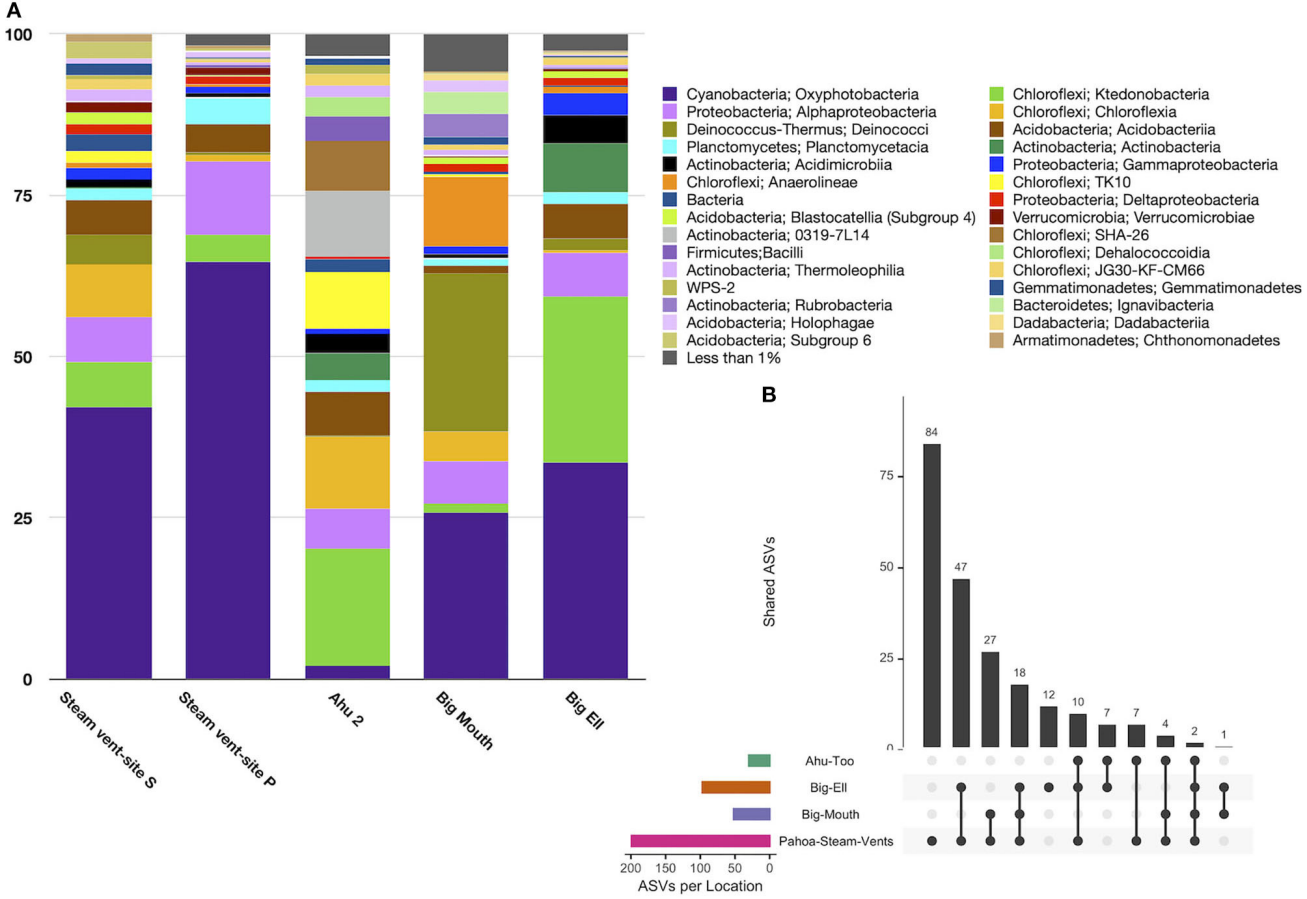
**(A)** Relative abundance by the site of class-level taxa with >1% abundance in geothermally active sites. Geothermal caves are dominated by Cyanobacteria (Oxyphotobacteria; 40.2% ± 25.3) and Chloroflexi including classes Ktedonobacteria (10.6% ± 15.9) and Chloroflexia (5.8 ± 9.8). Steam vent sites hosted the highest abundance of Oxyphotobacteria. Ktedonobacteria was higher in geothermal caves. Deinococci were most abundant in Kīlauea Caldera's Big Mouth cave. **(B)** Upset plot illustrating overlapping ASVs among geothermal sites. Steam vents hosted many unique ASVs (2,518), followed by Big Ell cave. This shows that geothermal sites are largely unique environments, with few overlapping ASVs; the highest number of overlapping ASVs was in Big Ell and Pahoa Steam vents, which may in part be due to the larger number of samples from these locations. Only one ASV occurred in all sites (Class Oxyphotobacteria, *Chlorogloeopsis* sp.).

Diversity in geothermal sites at the taxonomic level of the class was dominated by sequences identified as Oxyphotobacteria (40.2% ± 25.3), followed by Chloroflexi, class Ktedonobacteria (10.6% ± 15.9), and the Deinococcus-Thermus, class Deinococci (5.4% ± 9.4; Figure 3A). Geothermal sites hosted 103 taxonomic classes, with only five of those classes comprising >5% of the average relative abundance of ASVs. Eighty-seven classes were <1% of the average relative abundance. Figure 3B illustrates the overlap of ASVs among all geothermal sites, with 2,518 ASVs occurring only in Pahoa steam vents, while just one ASV (998bd9a9277837ead97ae349a5ec4df7; Class Oxyphotobacteria, *Chlorogloeopsis* sp.) was detected in all sites. Geothermal caves in Kīlauea Caldera had 826 unique ASVs in Big Ell cave alone, 394 in Big Mouth, and 184 in Ahu Too. No ASV occurred in all three of these caves.

**Figure 4:**
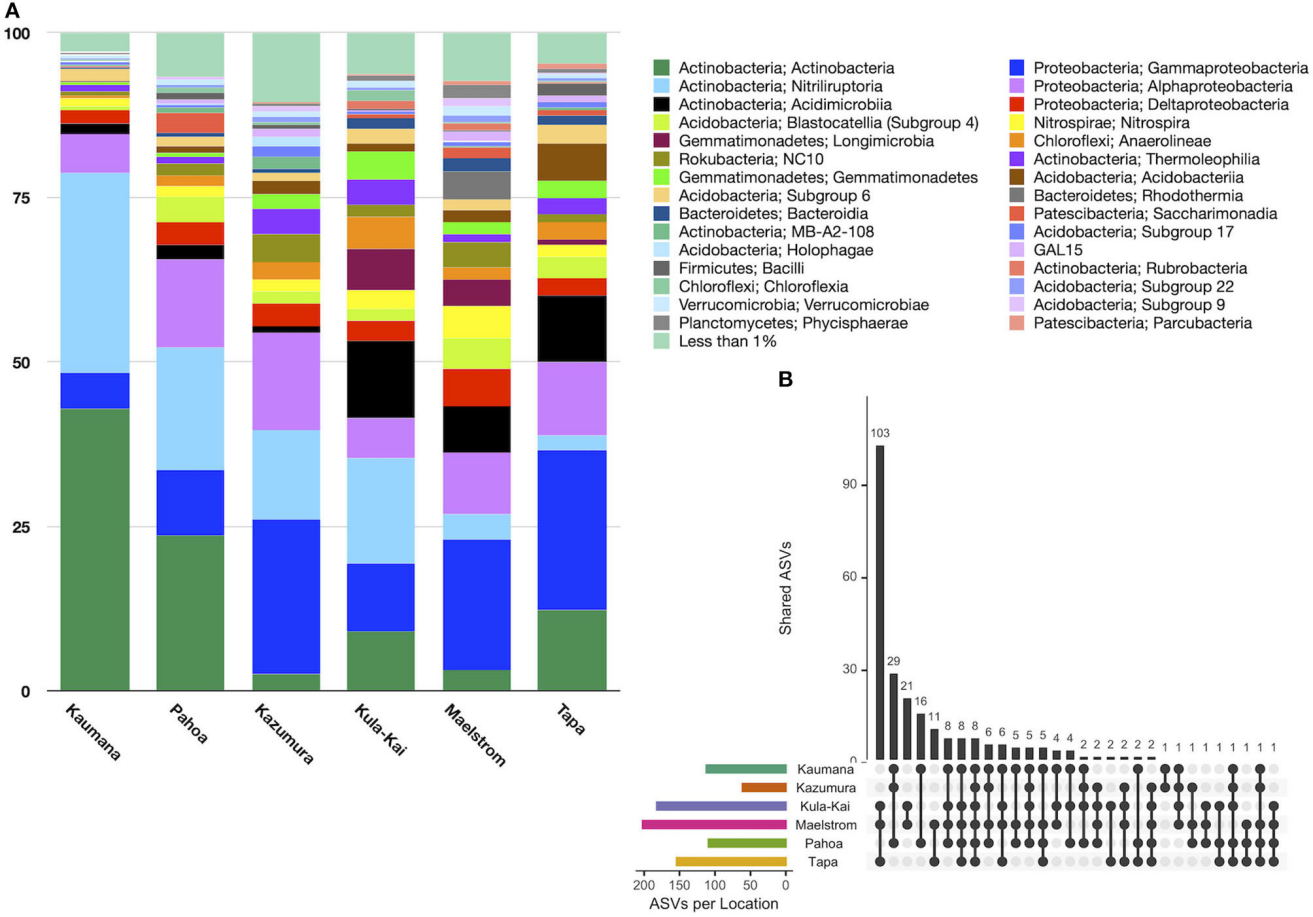
**(A)** Relative abundance by the site of class-level taxa with >1% abundance in lava tubes. Dominant taxa in these features were Actinobacteria (16.0% ± 24.4), Gammaproteobacteria (15.4% ± 11.0), Nitriliruptoria (13.7% ± 23.3), and Alphaproteobacteria (9.8% ± 10.7). Longimicrobia and Acidimicrobiia ASVs were considerably more abundant in the Kipuka Kanohina cave system in the south of the island of Hawai‘i. **(B)** Upset plot of lava tube sites shows that individual lava tubes are moderately unique, with Maelstrom cave hosting 1,277 unique ASVs, followed by Kaumana cave (1,022 unique ASVs). Only seven ASVs occurred in all lava tubes.

In total, 151 taxonomic classes were represented in lava tube samples, with three classes on average comprising >10% of the relative abundance, including Actinobacteria (16.0% ± 24.4), Gammaproteobacteria (15.4% ± 11.0), and Nitriliruptoria (13.7% ± 23.3; Figure 4A). Alphaproteobacteria were also common, with a relative abundance of 9.3% (±5.3). Variation across samples was high, and no one class was dominated in all lava tubes. Only six classes were represented by mean relative abundances >5%. Conversely, 137 classes were each represented by <1% of the mean relative abundance of ASVs.

An Upset plot of lava tube samples illustrates that all lava tubes had hundreds, and occasionally thousands of unique ASVs, while only seven ASVs occurred in all lava tubes (Figure 4B). Maelstrom, a part of the Kipuka Kanohina cave system, hosted the most unique ASVs (1,277), followed by Kāumana cave (1,022). Kula Kai and Maelstrom caves, part of the same larger cave system, shared the most ASVs (284).

### Phylogenetic Distinctness and Community Structure

**Figure 5:**
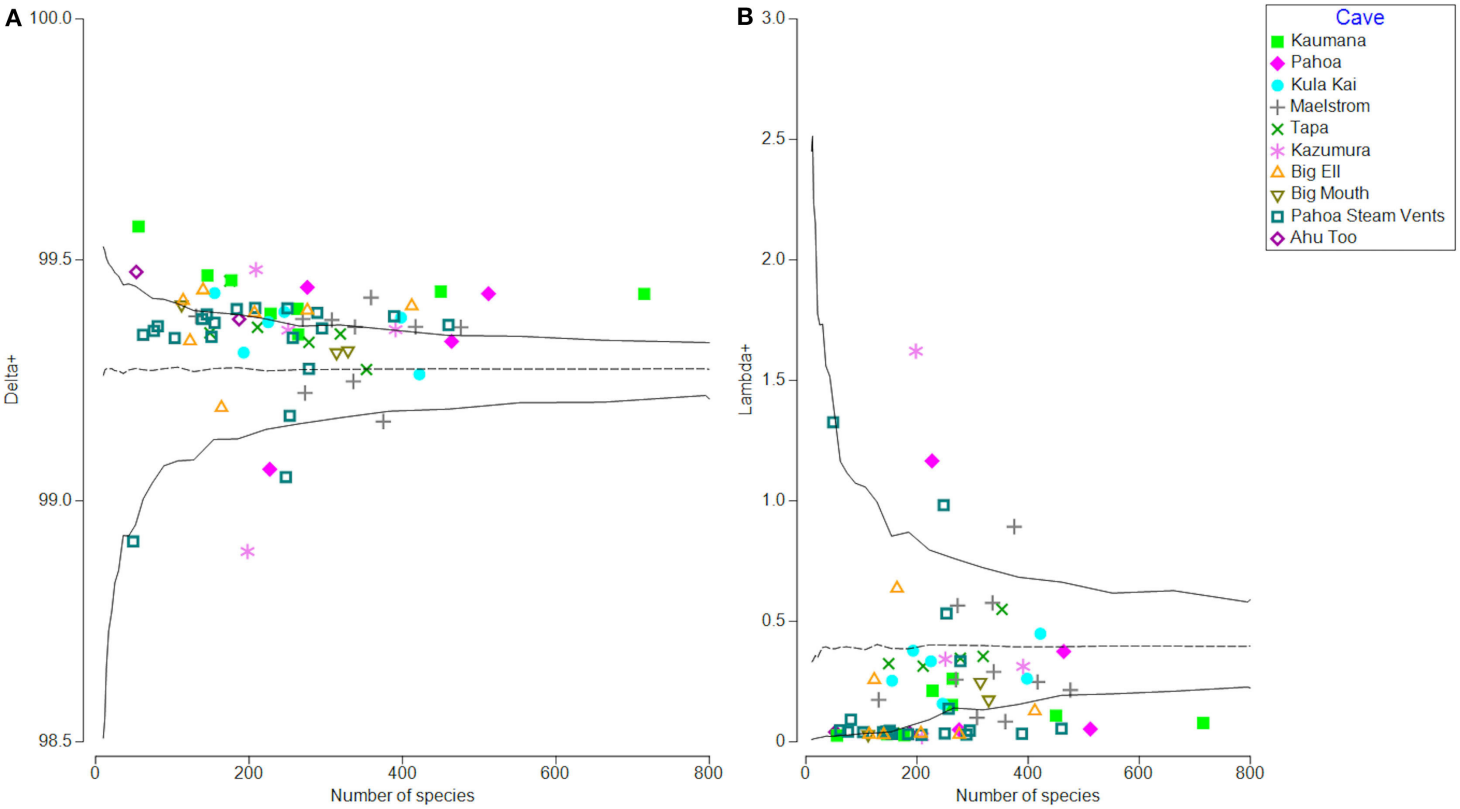
Phylogenetic distinctness (Delta+) on the left **(A)** and variation of phylogenetic distinctness (Lambda+) on the right **(B)** for all samples based on patristic distances between identified taxa (ASVs). Samples are labeled by location. Most samples are either within expected values of Delta+ (null = randomly distributed) or higher than expected, meaning samples are phylogenetically over-dispersed, possibly due to competitive exclusion **(A)**. Variation in phylogenetic distinctness (Lambda+) is within expected values or lower than expected. Most samples had lower than expected variation in distances between branches on the phylogenetic tree, when compared to the null model of random assemblage, and represent that more distinctly related organisms are assembled into the community **(B)**.

Phylogenetic community structure in all 70 samples was investigated through average phylogenetic distinctness (Delta+ and Lambda +; Clarke and Warwick, 1998), which provides a framework to test departures from expectations from a null model through permutation tests using the TAXDTEST (PRIMER-e v7.0). This algorithm tests the null hypothesis with which the community composition and structure are randomly assembled. Figure 5 illustrates that within lava tubes and geothermal sites, most samples had higher than expected average phylogenetic distinctness (Delta+; Figure 5A). The variance of phylogenetic distinctness was below what would be expected by chance (Lambda+; Figure 5B). This suggests that the breadth of phylogenetic diversity is higher in lava caves and geothermal sites than if community assemblages were structured randomly, with greater phylogenetic distances between ASVs than if communities were assembled randomly (i.e., over-dispersed). This pattern is often driven by competitive exclusion in oligotrophic environments (Begon et al., 1998; Barton, 2015), Five samples had lower than expected Delta+ and higher than expected Lambda+ values, suggesting habitat filtering may structure the communities in those samples, with ASVs having less phylogenetic distance than would be expected if communities were assembled randomly. Four of these samples were biofilm or microbial mats from different locations, along with a single blue-green mineral sample collected from the Maelstrom cave.

### Network Analyses

**Figure 6:**
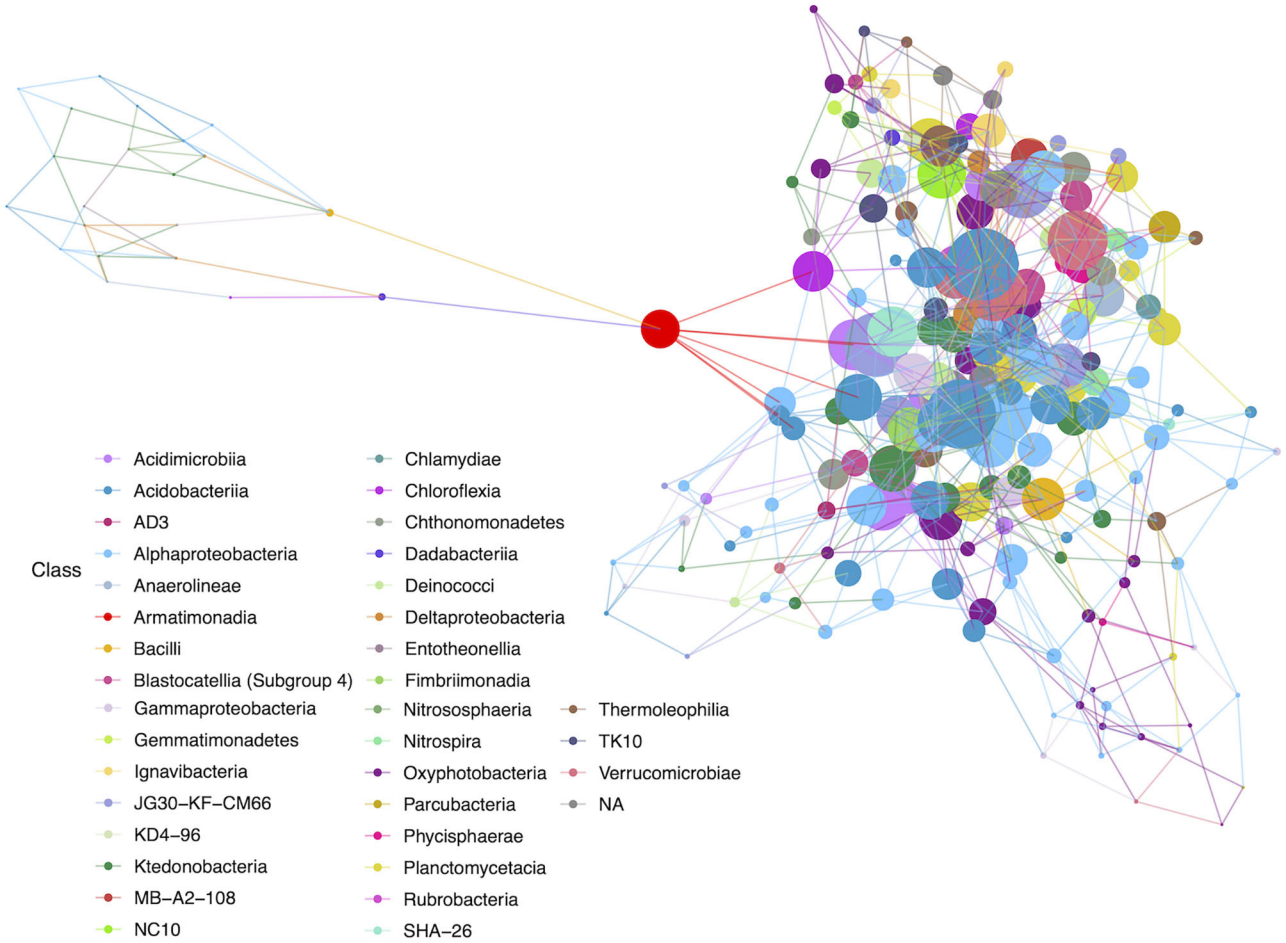
Hub network for geothermal sites, illustrating a high level of complexity within the network. There are 100 nodes that have an above-average hub score (represented by larger circles in the network). Hubs are taxa that most likely have important ecological roles, such as keystone species, and help maintain the overall network structure.

Network analyses were completed to identify community interactions and microbial co-occurrence at the class level. Networks were analyzed separately for geothermal sites and lava tubes due to a lack of overlapping among ASVs in both environments (refer to Figures 6, **9**). Table 1 provides the overall network statistics for each environment. Geothermal sites had a greater number of edges (connections) than lava tubes, even though there were more nodes (ASVs) included in the lava tube network. Network density, or the ratio of edges to nodes, was, therefore, higher in geothermal sites (Table 1; network density = 3.078). The average degree of centrality (the average number of edges for a given node) was also higher in the geothermal network (Table 1).

**Table 1:** Network statistics comparing geothermal sites to lava tubes.

**Environment**	**# Nodes**	**# Edges**	**Avg. degree centrality**	**Avg. betweenness**	**Avg. closeness**	**# of edges/# of nodes**
Geothermal sites	218	671	6.16	309.56	0.0012	3.078
Lava tubes	259	521	4.02	576.80	0.0006	2.012

*Geothermal sites had a greater number of edges, a higher average degree of centrality, and a higher average hub score. The average betweenness centrality also was higher in lava tubes and suggests that interactions are greater in geothermal sites than in lava tubes. # Nodes, total number of ASVs in the network; # Edges, total number of connections (edges) between nodes; Avg. degree centrality, average number of edges in a network that connects to one node; Avg. betweenness, betweenness centrality measures the extent to which a node lies on paths between other nodes and can be used to identify which ASVs interact most with other members of the community network; Avg. closeness, average closeness centrality which measures how far a node is to all other nodes and can be used to find the most central taxa of a given community network; # edges/# nodes provides network density*.

**Figure 7:**
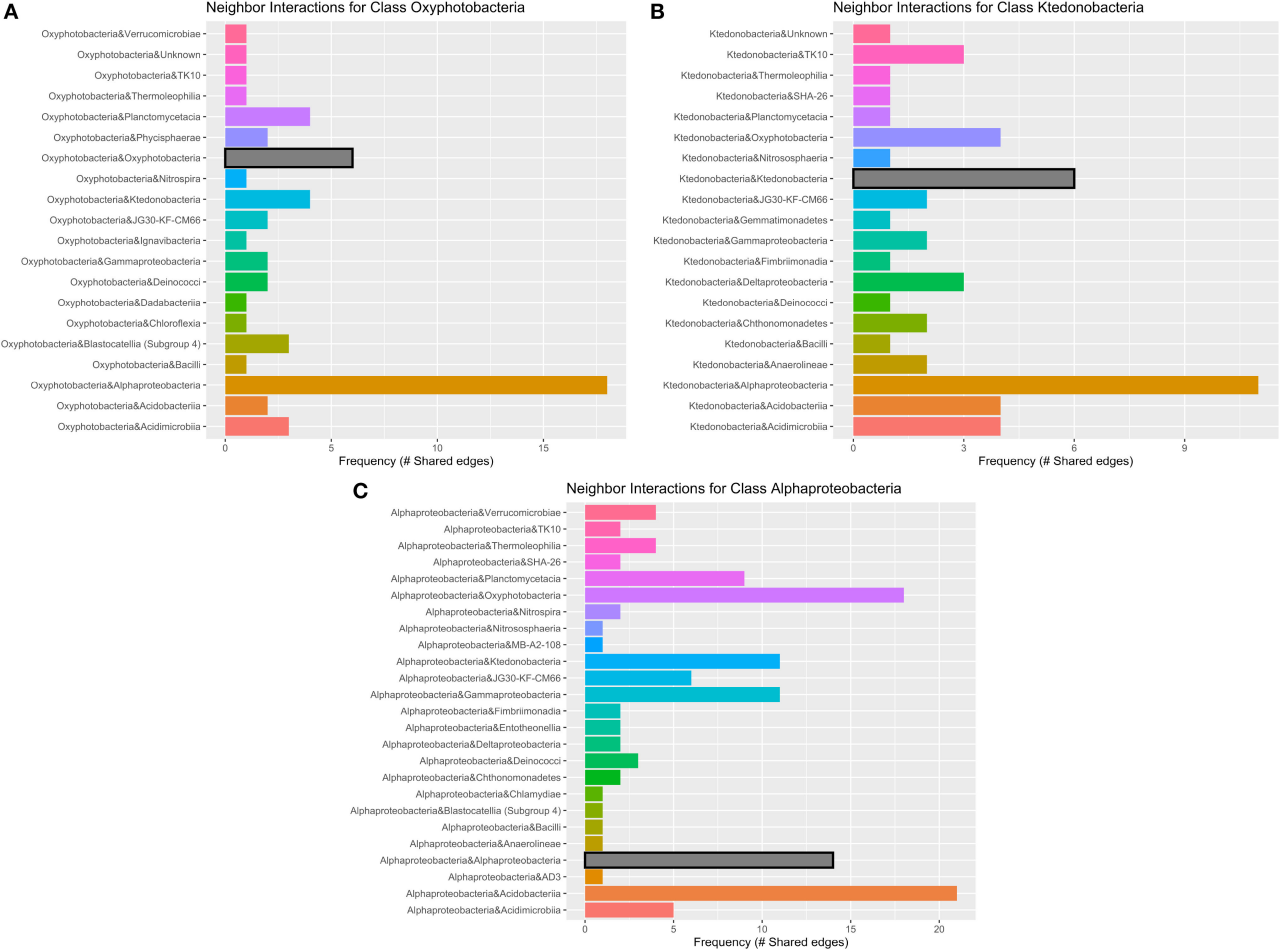
Geothermal neighbor interaction frequency plots at the class level of the three classes with the greatest number of interactions (edges) within the geothermal network: **(A)** Oxyphotobacteria, **(B)** Alphaproteobacteria, and **(C)** Ktedonobacteria. Oxyphotobacteria have the largest number of interactions or co-occurrence with Alphaproteobacteria. However, Alphaproteobacteria have the most interactions with Acidobacteriia, followed by Oxyproteobacteria and then other Alphaproteobacteria. Ktedonobacteria, a common Chloroflexi group in geothermal sites, also has the greatest number of interactions with Alphaproteobacteria, followed by interactions with other Ktedonobacteria.

Among the geothermal sites, a network was constructed using a filter threshold of ≥12.5% taxa from all geothermal samples. Figure 6 illustrates the network constructed with hubs identified, while Figure 7 provides neighbor interaction bar graphs, illustrating which classes had the greatest number of co-occurrence relationships (edges) in geothermal sites. There are 218 nodes in the geothermal network, with each node representing a single ASV identified at the class level and sized by the node's hub score. Hub scores are scaled from 0 to 1 and cannot be directly compared between networks but provide information on which nodes have the greatest number of connections and betweenness centrality, and therefore, the removal of those hubs causes the collapse of parts of a network.

There are 671 edges (positive interactions) in the geothermal network. The average number of connections to any one ASV was 6.16 (degree centrality; Table 1). The classes with the greatest number of co-occurrence interactions were Oxyphotobacteria, Alphaproteobacteria, and Ktedonobacteria (Figure 7). Consistently, taxa had more connections with other ASVs that were distantly related to taxa, rather than interactions with ASVs within the same phyla or class. Oxyphotobacteria had the most interactions with Alphaproteobacteria, while Alphaproteobacteria had the greatest number of interactions with Acidobacteriia, a class of Acidobacteria (Figure 7). Ktedonobacteria, the other taxa with a relatively high abundance in many geothermal samples, had the greatest number of interactions with Alphaproteobacteria as well (Figure 7).

The ASV with the highest hub score was affiliated with the class Acidobacteriia (hub score = 1; Supplementary Table 4), while another Acidobacteriia (Subgroup 2) affiliated sequence had the second-highest hub score (0.961). These ASVs may be derived from organisms with potentially important ecological roles. An ASV identified as a Chloroflexi (class JG30-KF-CM66) had the third-highest hub score in the overall network (0.884). There were 100 ASVs with above average hub scores (Supplementary Table 4). Of those, 18% of ASVs affiliated with members of the Chloroflexi, mostly Ktedonobacteria, and 16% affiliated with Alphaproteobacteria, mostly Rhizobiales. Sequences affiliated with Oxyphotobacteria were the most abundant in geothermal samples, but only six ASVs (6%) were identified with above-average hub scores. Acidobacteriia-affiliated ASVs comprised 17% of the ASVs with above-average hub scores. Most of the Acidobacteriia ASVs affiliated with the Solibacteraceae (Subgroup 3), including three that were most closely identified with a *Bryobacter* sp.

Seven subnetworks (consortia) were identified and constructed from geothermal sites. Table 2 provides information on all seven consortia, the dominant phyla, and the taxonomic identification of the ASV with the highest hub score (refer to Supplementary Figure 5 for images of all geothermal subnetworks and Supplementary Table 5 for subnetwork statistics and hub scores). A phylogenetic tree of all ASVs in geothermal subnetworks is illustrated in Supplementary Figure 6, highlighting the taxonomy of ASVs that occurred in more than one consortium, and ASVs that had the highest hub score within each of the seven subnetworks.

**Table 2:** Subnetwork statistics and information for geothermal sites.

**Subnetwork no**.	**Nodes**	**Edges**	**Edge density**	**Network density**	**Most abundant phyla**	**Highest hub score**
Consortia 1	29	63	0.155	2.172	Proteobacteria (24.1%)	Phycisphaerae
Consortia 2	45	118	0.119	2.622	Chloroflexi (24.4%)	Thermoleophilia
Consortia 3	17	29	0.213	1.706	Chloroflexi (41.2%)	Ktedonobacteria
Consortia 4	53	116	0.0842	2.189	Proteobacteria (34.0%)	*Hyphomicrobium* sp.
Consortia 5	21	41	0.195	1.952	Proteobacteria (38.1%)	Xanthobacteraceae
Consortia 6	14	26	0.286	1.857	Proteobacteria (50%)	Elsterales
Consortia 7	39	95	0.128	2.435	Acidobacteria (28.2%)	Acidobacteriales

*Edge density is defined as the number of actual edges/maximum possible edges. Network density is defined as the number of edges/number of nodes and expresses the level of modularity of a subnetwork. The most abundant taxa occurring in each subnetwork, as well as the nodes (ASVs) with the top two highest hub scores are provided*.

In general, geothermal subnetworks comprised ASVs that were affiliated with a wide range of phyla and classes, but Chloroflexi and Proteobacteria-affiliated ASVs were the most abundant except in consortia 7 which was dominated by Acidobacteria-affiliated ASVs (Table 2). Although there was a wide range of phyla and classes in consortia, there were often ASVs that were identified as the same family or genus in multiple consortia (Supplementary Figure 6; i.e., multiple branches of Gemmataceae, *Bryobacter* sp., etc.). In all but one geothermal subnetwork, ASVs identified as the deep-branching phylum Chloroflexi had above-average hub scores, and in one consortium, a Chloroflexi-affiliated ASV had the highest hub scores (Table 2). This suggests they have high levels of interactions and may occupy an important ecological niche. Among the Chloroflexi, many were classified as yet-uncultured classes, such as JG30-KF-CM66, Anaerolineae, and Ktedonobacteria, with Consortia 2 and 3 containing the most Chloroflexi-affiliated sequences (Table 2). ASVs classified as Ktedonobacteria (order B10-SB3A) and JG30-KF-CM66 had the top two hub scores in that subnetwork (Supplementary Table 5).

Oxyphotobacteria were the most abundant taxa in geothermal samples, and 13 nodes in Consortium 4 comprised Oxyphotobacteria-affiliated ASVs. These co-occur with 18 Alphaproteobacteria-affiliated nodes, the other most abundant taxa identified in consortium 4 (Supplementary Table 5). Overall, 19 ASVs categorized as Oxyphotobacteria appeared in all subnetworks, but 68% of those occurred in one subnetwork, consortium 4 (Supplementary Figure 5 and Supplementary Table 5).

Proteobacteria-affiliated ASVs were the most abundant across all geothermal subnetworks, representing 46 branches in the sub-network phylogenetic tree (Supplementary Figure 6). Proteobacteria were also among the top hub scores in consortia 4–6. A Proteobacteria ASV, affiliated with the genus *Pedomicrobium* sp., was detected across five different consortia, the most of any ASV, but it did not have high hub scores (Supplementary Figure 6). Members of this genus are known for biofilm formation and manganese or iron oxidation (Larsen et al., 1999).

**Figure 8:**
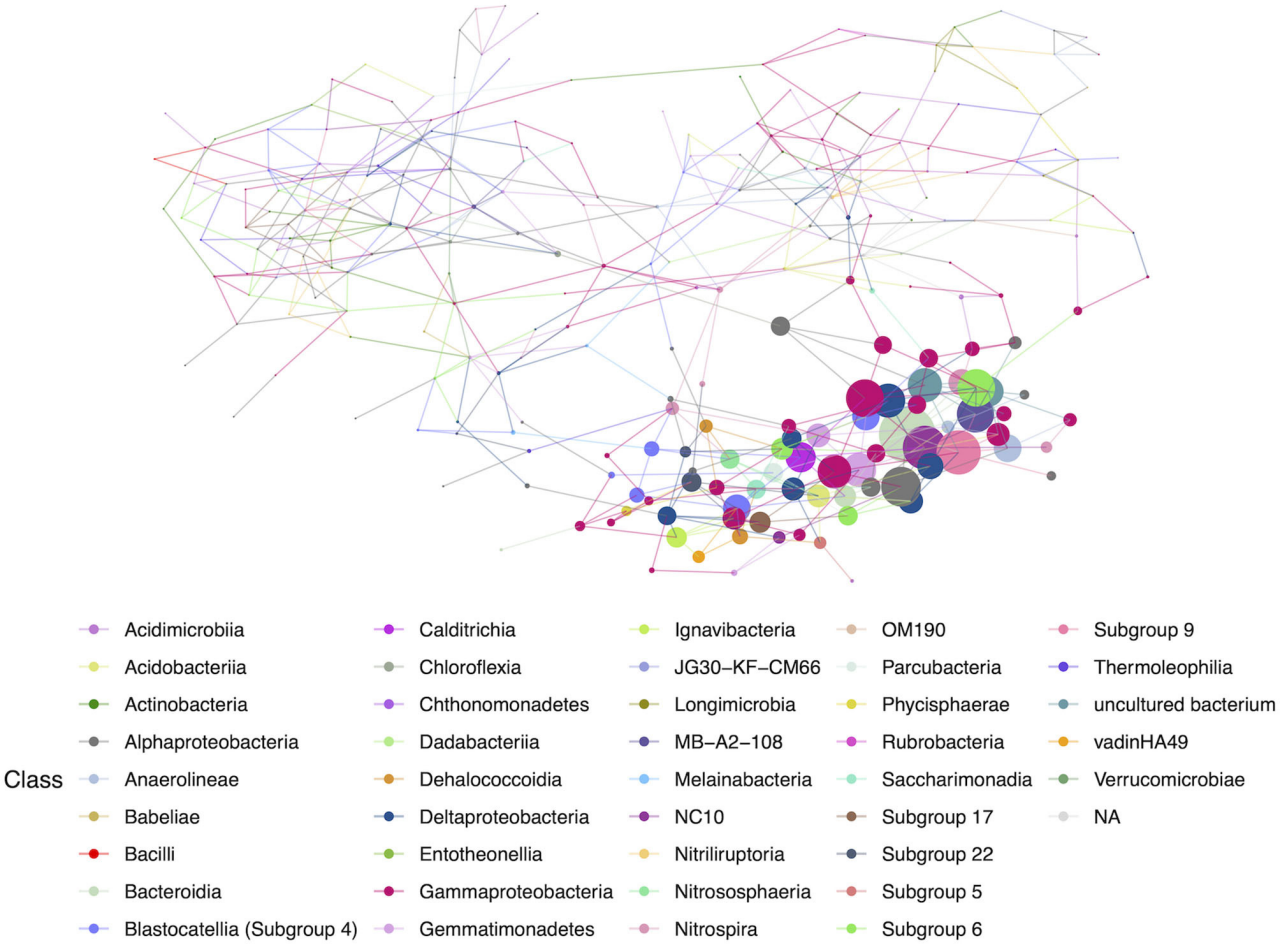
Hub network for lava tubes, suggesting lower complexity than the geothermal network. There are 69 ASVs with an above-average hub score, represented by larger circles on the network graph. Hubs are taxa that most likely have important ecological roles, such as keystone species, and help maintain the overall network structure.

**Figure 9:**
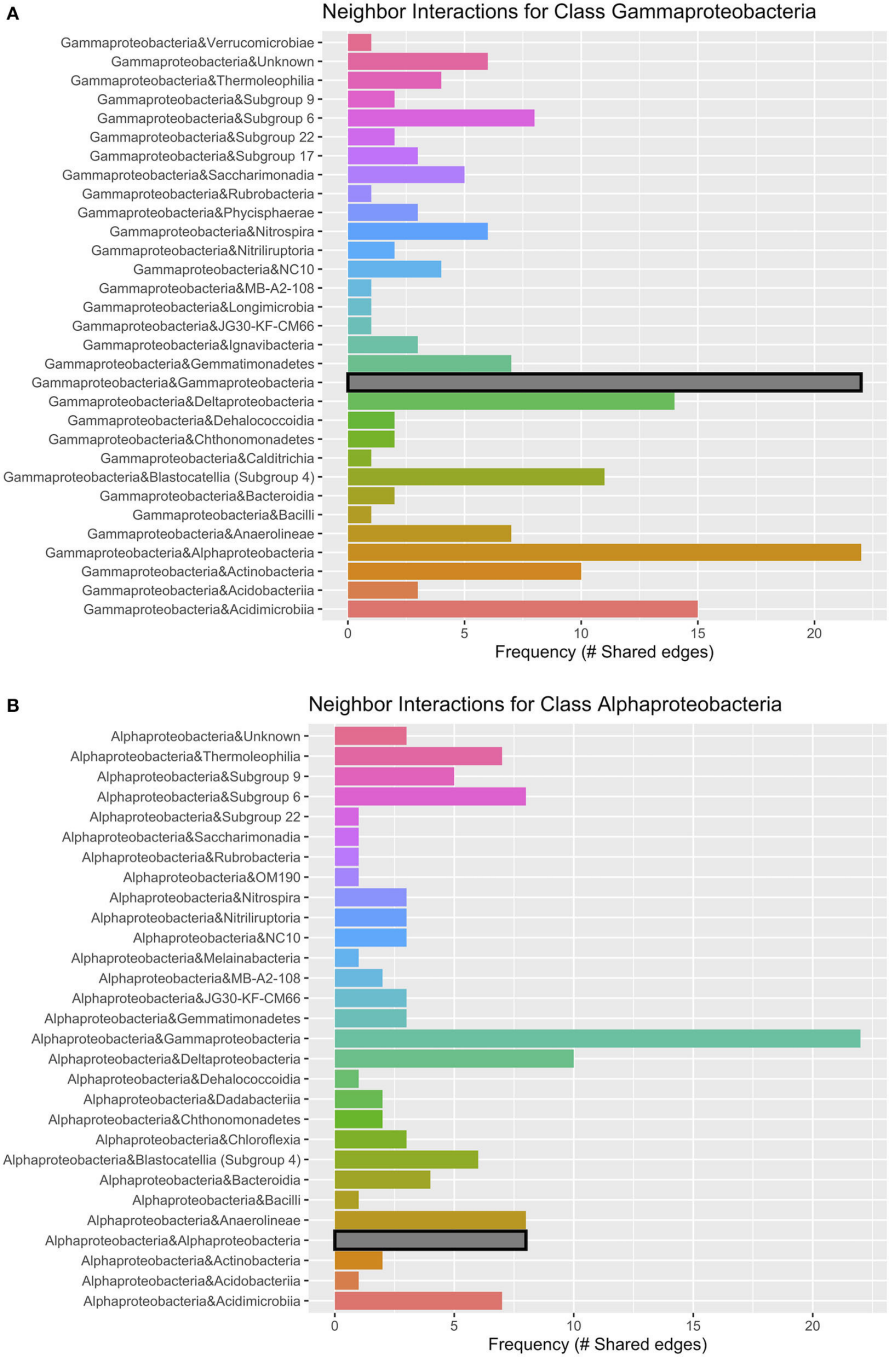
Lava tube neighbor interaction frequency plots at the class level of the two classes with the greatest number of interactions (edges) within the network: **(A)** Gammaproteobacteria and **(B)** Alphaproteobacteria. Gammaproteobacteria have the highest frequencies of neighbor interactions with other Gammaproteobacteria, as well as Alphaproteobacteria. Gammaproteobacteria also interact or co-occur with Acidimicrobiia (phylum Actinobacteria), and Deltaproteobacteria. Alphaproteobacteria co-occur with Gammaproteobacteria at the greatest frequency and have much fewer interactions with other Alphaproteobacteria. Deltaproteobacteria and Chloroflexi class Anaerolineae also had a high frequency of interactions with Alphaproteobacteria within lava tubes.

Networks constructed for the lava tube samples used a threshold cutoff of 15% sample prevalence, and comprised 251 nodes and 521 edges, with an average degree centrality of 4.02 (Figure 8; Table 1), lower than geothermal sites, suggesting a less interactive environment overall. In addition, unlike geothermal sites, ASVs from lava tubes tended to have a greater number of interactions with ASVs in the same phylum or class (Figure 9). For example, Gammaproteobacteria and Alphaproteobacteria had the highest counts of neighbor interactions in both Proteobacteria classes (Figure 9). Gammaproteobacteria interacted with other Gammaproteobacteria, and also had a high number of interactions with Alphaproteobacteria and Acidomicrobiia. Alphaproteobacteria formed the most interactions with Gammaproteobacteria, followed by Deltaproteobacteria.

An ASV associated with the class Bacteroidia (*Microscillaceae* sp.) had the highest hub score in the overall lava tube network (hub score = 1), followed by an Acidobacteria (class Subgroup 9; hub score = 0.77), and a Rokubacteria (*Candidatus Methylomirabilis* sp.; hub score = 0.766; Supplementary Table 6). There were 69 ASVs with an above-average hub score, and 46.4% of those were affiliated with Proteobacteria. Nineteen of the ASVs with above-average hub scores were identified as Gammaproteobacteria (27.5%), seven were Alphaproteobacteria (10.1%), and six were Deltaproteobacteria (8.7%; Supplementary Table 6). This illustrates that in lava tubes, the phylum Proteobacteria dominates in terms of the number of interactions. Acidobacteria also had a large percentage of the above-average hub scores (20.2% of ASVs). Although Actinobacteria are commonly cultured from caves and had the highest relative abundance among lava tube samples, only one ASV identified as an Actinobacteria (class MB-A2-108) was found to have a higher-than-average hub score in the overall lava tube network (hub score = 0.646, which was the 7th highest hub score; Supplementary Table 6).

Eight subnetworks were constructed from the lava tube data set (Table 3; Supplementary Figure 7 and Supplementary Table 6). A phylogenetic tree was constructed using only the ASVs within lava tube subnetworks, with Proteobacteria having the most ASVs identified in more than one consortium (Supplementary Figure 8). Within the Proteobacteria, five ASVs identified as Gammaproteobacteria occurred in more than one subnetwork. However, there was no ASV that occurred repeatedly in most subnetworks in lava tubes, unlike geothermal sites (Supplementary Figure 6; i.e., *Pedomicrobium* sp.). The majority of ASVs with top hub scores in subnetworks were affiliated with Proteobacteria, Acidobacteria, Actinobacteria, Gemmatimonadetes, and the phyla, GAL15 (Table 3). Candidate phylum, GAL15 has been recovered from subsurface soil communities previously (Brewer et al., 2019), and in the carbonate cave, Fort Stanton Cave (Kimble et al., 2018). Within Acidobacteria, ASVs associated with Blastocatellia (Subgroup 4) were common in lava tube consortia and had top hub scores in four of the subnetworks (Table 3; Supplementary Figure 7 and Supplementary Table 6), and therefore may be species of ecological importance.

**Table 3:** Subnetwork statistics and information for lava tubes.

**Subnetwork no**.	**Nodes**	**Edges**	**Edge density**	**Network density**	**Most abundant phyla**	**Highest hub score**
Consortia 1	28	40	0.106	1.429	Actinobacteria (32.1%)	Pyrinomonadaceae
Consortia 2	64	120	0.0596	1.875	Proteobacteria (34.9%)	Hyphomicrobiaceae
Consortia 3	34	57	0.102	1.677	Proteobacteria (41.2%)	Betaproteobacteriales
Consortia 4	13	20	0.256	1.538	Actinobacteria (30.8%) and Proteobacteria (30.8%)	Nitriliruptoraceae
Consortia 5	54	110	0.077	2.037	Proteobacteria (44.4%)	Blastocatellia (Subgroup 4)
Consortia 6	16	19	0.158	1.188	Proteobacteria (56.3%)	Acidimicrobiia (order IMCC26256)
Consortia 7	23	35	0.138	1.522	Proteobacteria (62.5%)	Gemmatimonadaceae
Consortia 8	26	46	0.142	1.769	Proteobacteria (50%)	GAL15

*Edge density is defined as the number of actual edges/maximum possible edges. Network density is defined as the number of edges/number of nodes and expresses the level of modularity of a subnetwork. The most abundant taxa occurring in each subnetwork, as well as the nodes (ASVs) with the top highest hub score are provided*.

## Discussion

### Lava Tubes and Geothermal Sites Harbor Unique Microbial Communities

Lava tubes, geothermal caves, and fumaroles on the island of Hawai‘i contain unique microbial ecosystems with little overlap between caves or sites. Samples described here were collected over multiple visits spanning a decade, but no significant association was detected between diversity patterns and time of collection. In all samples, most ASVs were not able to be assigned with high confidence to a named genus or species, but were microbial “dark matter,” those unidentified at the lower taxonomic levels of family or genus (Zamkovaya et al., 2020). This suggests that caves and fumaroles are under-explored diverse ecosystems. Moreover, the bacterial communities defined here are likely to be structured by competitive exclusion arising from low nutrient availability, creating a phylogenetic pattern with a broader range of taxa than would be expected by chance in most samples (over-dispersion). This may explain the relatively high diversity observed in Hawaiian lava caves and fumaroles, and potentially other locations around the world (Northup and Lavoie, 2015; Riquelme et al., 2015; Wall et al., 2015).

Consortia in lava caves and geothermal sites may be unique groups of microorganisms that reflect the small-scale spatial variations in mineral composition of basalts and other micro-environmental variables. However, network analysis, including the identification of subnetworks, suggests that there are several closely related ASVs that occur in multiple consortia. This is observed in Supplementary Figures 6, 8, which show the phylogenetic relationships of ASVs identified in only subnetworks, and which subnetworks each ASV. Although there is a considerable diversity at higher taxonomic levels, within several classes, ASVs identified as the same order, family, or genus are found repeatedly (i.e., *Bryobacter* sp., in geothermal sites and Gemmatimonadaceae in lava caves). These may be the same organism or have the same functional role within consortia, but this information is unknown and cannot be defined using methods that target 16S rRNA fragments. Sequencing technology that generates longer sequences (i.e., MinION or PacBio), culture-based methods that target specific taxonomic groups, and metagenomic sequencing could help determine the identity of these organisms within consortia in Hawaiian volcanic environments, as well as what their ecological roles are likely in these communities.

### Phylogenetic Diversity Is Greater in Lava Tubes and Older Caves

Geothermally active sites in Hawai‘i, including lava caves in Kīlauea Caldera and fumaroles, host different bacterial communities than lava tubes. In general, phylogenetic diversity in geothermal sites, defined here by Faith's PD, was statistically lower than in lava tubes and was dominated by ASVs that were affiliated with Cyanobacteria and Chloroflexi. Lava tube bacterial communities were dominated by Actinobacteria and Proteobacteria-affiliated ASVs. However, Kaumana cave, a lava tube that is only ~130 years old had lower phylogenetic diversity than other lava tubes, similar to geothermal caves and fumaroles that occurred in younger lava flows. We posit that this reflects the age of the basalt, consistent with previous studies that also concluded diversity was lower in younger basalts (King, 2007). Our data illustrate that caves aged 500–800 years old host greater phylogenetic diversity than sites that are 65–400 years old. Microbial communities in geothermal sites and lava tubes can likely be placed on a spectrum of diversity over time, with those in geothermal sites representative of early stages of microbial colonization on basalts and in areas of volcanic activity. As these communities age and/or cool, Proteobacteria and Actinobacteria may become prevalent.

### Hawaiian Volcanic Features Are Phylogenetically Over-Dispersed

Geothermal sites and lava tubes have distinct phylogenetic signatures of over-dispersion, a phylogenetic community structure that has not been commonly documented in microbial communities (Lozupone and Knight, 2008). In many ecosystems, phylogenetic over-dispersion occurs when competitive exclusion is a driving factor in structuring communities (Webb, 2000; Lozupone and Knight, 2008). Competitive exclusion is more common in oligotrophic environments, such as caves and fumaroles, where there is strong competition for limited resources. In few studies using null models to determine a phylogenetic signature of microbial communities, and the possible underlying stochastic and deterministic processes, habitat filtering (phylogenetic clustering) was observed most often (Horner-Devine and Bohannan, 2006; Aguirre De Cárcer, 2019). Phylogenetic clustering occurs when closely related bacterial species share a trait, or suite of traits, that allow them to persist in a given habitat. However, our data do not support habitat filtering as the major factor structuring microbial community assemblies in lava caves and fumaroles, but instead, support high diversity driven by competitive exclusion.

In networks constructed from geothermal samples, phylogenetically similar species do not co-occur together, and instead, more distantly related species co-occur, which also suggests that community assemblages are being driven by competitive interactions between closely related species that eliminate or reduce closely related species in the samples. The exception was Gammaproteobacteria within lava tubes, which have a higher frequency of interactions with other Gammaproteobacteria and Alphaproteobacteria. In lava tube networks, Proteobacteria interacted more with other Proteobacteria, unlike in geothermal sites. However, only five samples out of 70 had a phylogenetically clustered signature, but those five samples were not from any one location or sample type, and we could not determine a specific environmental reason that would explain the difference in pattern.

### The Complexity of Bacterial Networks Is Higher in Geothermal Sites

Network analyses concluded that the overall complexity of interactions among bacteria is higher in geothermal environments than in lava caves. Bendia et al. (2021) reported a similar pattern of greater complexity in microbial communities along a thermal gradient. This observation may reflect the more extreme nature of the environment and lower diversity overall, a situation in which more synergistic metabolic interactions may be required for survival, with fewer individual strains or species able to fulfill the required ecological roles (greater number of hubs). Geothermal subnetworks were also more complex (higher number of nodes and edges) than those in lava tubes. These findings did not support our hypothesis that complexity would increase over time in volcanic environments as niche-specific consortia develop in the more stable environments of lava tubes.

Higher overall diversity occurred in lava tube samples, but the complexity of interactions was less in lava tubes. Moreover, the most abundant ASVs in these communities were not hubs in the overall networks or the subnetworks. This is important for subsequent investigations of the cave and fumarole environments, which should use culturing methods combined with sequencing technologies to target these taxa with potentially important ecological roles in these unique environments.

Chloroflexi may play important ecological roles in communities both in lava tubes and geothermal sites, given they occurred in almost every subnetwork (consortium), and frequently with high hub scores. Some cultivated Chloroflexi are aerobic thermophiles, while others are anoxygenic photoautotrophs, for example, green non-sulfur bacteria that utilize low levels of light for photosynthesis. Such conditions are found in cave entrances in the “twilight zone” and at “skylights.” Therefore, ASVs identified as Chloroflexi may provide a carbon source through photoautotrophy in low light conditions. However, most of the Chloroflexi ASVs identified as hubs belong to groups with no cultured individuals and may have very different ecological roles in these understudied environments. Chloroflexi are also common in networks from lava tubes, where many of the samples would not have had any light. Further studies of these environments with a focus on Chloroflexi are needed to define their ecological roles in volcanic environments.

The most abundant groups, such as Oxyphotobacteria and Actinobacteria were not common in many consortia, and if they were present, they did not often have high hub scores. Therefore, the most abundant species may not have the most important ecological roles in these ecosystems, helping to create the overall stability of the community. Acidobacteria, another understudied group, commonly had the highest hub scores and is a group that requires further study to understand their ecological roles in caves and fumaroles. Cultivated members of Acidobacteria are considered aerobic oligotrophic bacteria due to their high abundances in low organic carbon environments (Kielak et al., 2016a) and were one of the five most numerous phyla in the Azorean lava caves (Riquelme et al., 2015). Others have been identified as acidophiles (Jones et al., 2009; Kielak et al., 2016a), which may suggest a possible reason these organisms were identified as “hubs.” Bacteria are capable of creating a low pH environment through the release of organic acids or by thriving in those low pH environments may be able to breakdown basalts and access nutrients in the rock through solubilization (Kielak et al., 2016b). However, Acidobacteria is likely a diverse group that requires further study to understand their important ecological roles as “hubs” in caves and fumaroles.

### Broader Context and Further Studies

Microbial communities associated with lava caves and fumaroles, representing a spectrum of environmental conditions commonly seen in volcanic environments, are of interest to a variety of fields and research beyond fundamental microbial ecology. In particular, Hawaiian lava tubes are of interest to astrobiology studies and upcoming missions to Mars (Boston et al., 2001; Northup et al., 2011; Bauermeister et al., 2014; Tarnas et al., 2021). Volcanic systems in Hawai‘i are geologically like those on ancient Mars, which had active volcanoes and fumaroles (Farmer, 1996; Thollot et al., 2012; Hynek et al., 2018; Sauro et al., 2020). High-resolution satellite images from various orbital spacecraft show that Martian volcanoes were built from countless individual flows, many of which were created through channels and lava tubes, signaling a style of volcanism analogous to Hawaiian eruptions (Sauro et al., 2020). With these geological similarities, Hawaiian volcanic environments can provide some insight into the possibility of life on Mars in its ancient past and how microbial communities could survive today on Mars in lava caves, or if introduced from Earth (forward contamination).

The study of cave and fumarole microbial communities is also important to questions in biotechnology, sustainable resource management, and bioremediation (Brune and Bayer, 2012; Zhang et al., 2016; Kapoore et al., 2022). Bioleaching organisms are commonly used in mining, and previous research has discovered that the recovery of target metals is often greater with co-cultures of chemolithotrophic organisms (Mathew and Krishnaswamy, 2017). Within the field of study focused on rare earth elements and biomining, the use and discovery of microbial consortia for higher rates of recovery of those elements are critical for more environmentally friendly mining methods (Fathollahzadeh et al., 2018). This also applies to space exploration and the potential to mine other planetary bodies and nearby asteroids (Cockell et al., 2020).

Understanding chemolithotrophic and acidophilic microbial consortia may also have importance in soil health and agriculture production, particularly in the desert or volcanic soils (Kielak et al., 2016b; Woo and Pepe, 2018). Chemolithothrophs and other organisms that bioweather basalts, are important in the breakdown of lava flows into soils, releasing nutrients into the biogeochemical cycle. Understanding these processes has become more important as we are faced with climate change and growing food concerns in areas with less water and low-nutrient soils than in previous decades. In addition, fungi play a very important role in bio-weathering in soils and rocks (Finlay et al., 2009), with one study suggesting that fungi may be responsible for ~40–50% of the breakdown of rock through bio-weathering (Li et al., 2016). Therefore, studies of cave systems and roles of microbes should include fungal communities, along with additional studies of rock–microbe interactions using geological methods (i.e., Wavelength-Dispersive X-Ray Spectroscopy, Ramen Spectroscopy, etc.). Additional studies that examine fungal–bacterial interactions in lava caves and geothermal sites are also needed to better define underlying mechanistic interactions in the bio-weathering process.

## Data Availability Statement

The datasets presented in this study can be found at Dryad: https://doi.org/10.5061/dryad.z612jm6f0, and with accession #PRJNA806456 in NCBI database.

## Author Contributions

RP completed most analyses and writing for this manuscript, the collection of samples in 2017, and DNA extractions. TZ completed network analyses and contributed largely to the interpretation and writing of the manuscript. SD and JS collected samples in 2006–2009 and 2019. DN and JM collected samples in 2017 and 2019. NM contributed to the analysis of samples through DNA extraction and molecular methods. JS, AD, and PC contributed funding for the project. RP, DN, and PB largely contributed to the ideas in this manuscript. All authors contributed to the editing and writing of the manuscript.

## Funding

This research was supported by the NASA Exobiology grant (80NSSC18K1064) and startup and University Facilitating Funds from George Washington University. This research was supported by funding from the NASA Exobiology Program (grant No. 80NSSC18K1064), startup and University Facilitating Funds from George Washington University and the University of Hawai'i at Mānoa; NSF Postdoctoral Research Fellowship in Biology (grant No. 1711856), and U.S. Department of Energy, Office of Science, Biological and Environmental Research Division (award number LANLF59T). This is contribution #167 from the School of Life Sciences at the University of Hawai'i at Mānoa.

## Conflict of Interest

The authors declare that the research was conducted in the absence of any commercial or financial relationships that could be construed as a potential conflict of interest.

## Publisher's Note

All claims expressed in this article are solely those of the authors and do not necessarily represent those of their affiliated organizations, or those of the publisher, the editors and the reviewers. Any product that may be evaluated in this article, or claim that may be made by its manufacturer, is not guaranteed or endorsed by the publisher.
