# Supplementary material for: Islands Within Islands: Bacterial Phylogenetic Structure and Consortia in Hawaiian Lava Caves and Fumaroles
Source: Front Microbiol. 2022 Jul 21;13:934708. doi: 10.3389/fmicb.2022.934708 (PMC9349362; doi:10.3389/fmicb.2022.934708)

Supplementary Figure 1. Rarefaction curves for all samples, both geothermal sites and lava tubes.

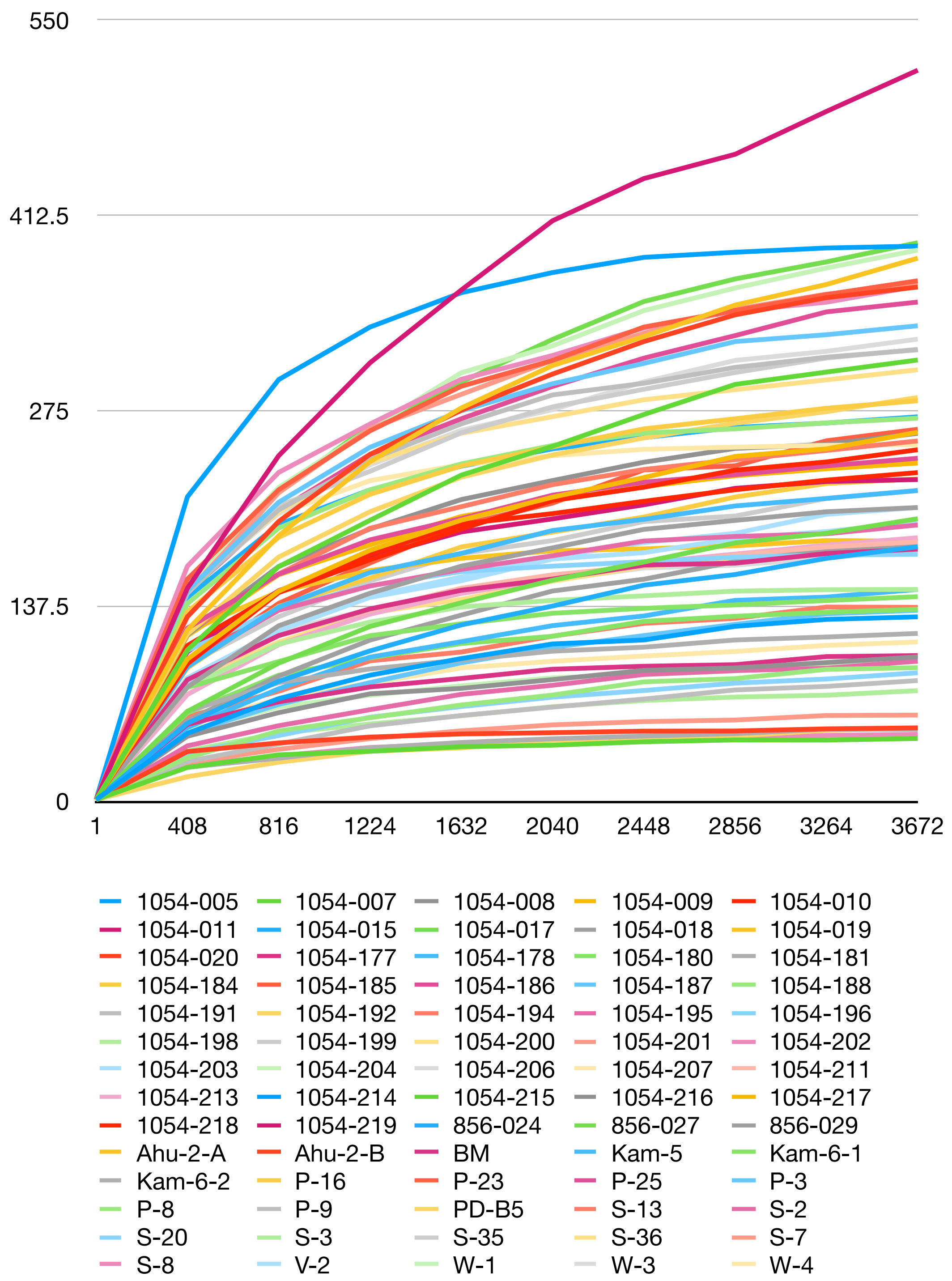

Supplement: Supplementary file 6 [file Data_Sheet_1.PDF]
