# Supplementary material for: Islands Within Islands: Bacterial Phylogenetic Structure and Consortia in Hawaiian Lava Caves and Fumaroles
Source: Front Microbiol. 2022 Jul 21;13:934708. doi: 10.3389/fmicb.2022.934708 (PMC9349362; doi:10.3389/fmicb.2022.934708)
Supplement: Supplementary file 7 [file Data_Sheet_2.PDF]

Supplementary Table 2: Kruskal-wallis-pairwise test of volcanic feature types

Group 1	Group 2	H	p-value	q-value
**Fumarole (n=20)**	Geothermal-cave (n=12)	0.03787878787879610	0.8456867367859360	0.8456867367859360
**Fumarole (n=20)**	lava-tube (n=38)	10.59794826048170	0.0011321322326197800	0.00204082848432998
**Geothermal-cave (n=12)**	lava-tube (n=38)	10.258513931888600	0.0013605523228866500	0.00204082848432998
