# Supplementary material for: Islands Within Islands: Bacterial Phylogenetic Structure and Consortia in Hawaiian Lava Caves and Fumaroles
Source: Front Microbiol. 2022 Jul 21;13:934708. doi: 10.3389/fmicb.2022.934708 (PMC9349362; doi:10.3389/fmicb.2022.934708)
Supplement: Supplementary file 8 [file Data_Sheet_3.PDF]

Supplementary table 2. Pairwise-test, Kruskal-Wallis statistical analysis of Faith's Phylogenetic Diversity (PD) vs. the estimated age of the lava flow for each site.

kruskal-wallis-pairwise-Cave-age

Group 1	Group 2	H	*p*-value	*q*-value
**100-years (n=12)**	130-years (n=8)	0.3809523809523730	0.5370939784426460	0.5967710871584960
**100-years (n=12)**	500-years (n=8)	9.05357142857143	0.0026218217690905900	0.006739945618996360
**100-years (n=12)**	65-400-years (n=20)	0.18333333333333700	0.6685241388865210	0.6685241388865210
**100-years (n=12)**	820-years (n=22)	9.82987012987013	0.0017170047166270200	0.006739945618996360
**130-years (n=8)**	500-years (n=8)	5.834558823529410	0.0157143497722189	0.026190582953698200
**130-years (n=8)**	65-400-years (n=20)	0.5068965517241400	0.476485410064148	0.595606762580185
**130-years (n=8)**	820-years (n=22)	5.94721407624634	0.014740564247431900	0.026190582953698200
**500-years (n=8)**	65-400-years (n=20)	9.002586206896550	0.0026959782475985400	0.006739945618996360
**500-years (n=8)**	820-years (n=22)	1.2668621700879800	0.260356161037775	0.3719373729111070
**65-400-years (n=20)**	820-years (n=22)	13.519661733615200	0.00023607696552450300	0.002360769655245030
