# Supplementary material for: Islands Within Islands: Bacterial Phylogenetic Structure and Consortia in Hawaiian Lava Caves and Fumaroles
Source: Front Microbiol. 2022 Jul 21;13:934708. doi: 10.3389/fmicb.2022.934708 (PMC9349362; doi:10.3389/fmicb.2022.934708)

Consortia #1

Consortia #1 consists of 29 members. Alphaproteobacteria make up 20.6 % of the ASVs. A Phycisphaerae identified as order Tepidisphaerales had the highest hub score. Two Verrucomicrobiae (family Chthoniobacteraceae), an Anaerolineae, and a Armatimonadetes had the top hub scores. Two Oxyphotobacteria, one of the most dominant groups abundance-wise also appear in this consortia, but did not have above average hub scores.

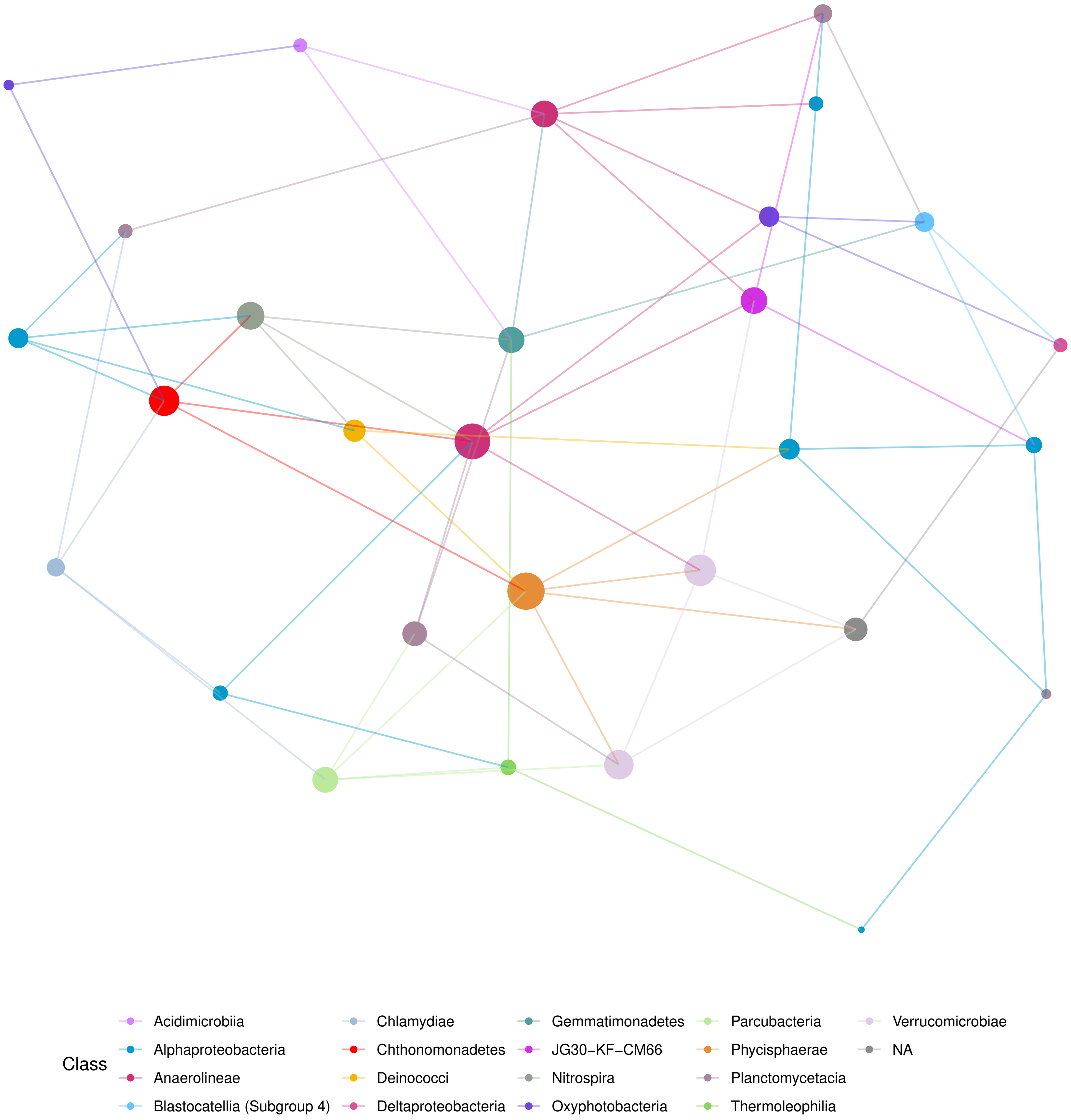

Consortia #2

Consortia # 2 consists of 45 members. Chloroflexi make up 24.4 % of the ASVs. An Actinobacteria belonging to class Thermoleophilia (Gaiellales) had the highest hub score.A Rokubacteria (class NC10), Planctomycetacia (Gemmataceae), Chlorflexi (class JG30-KF-CM66), and a Rubrobacteria (*Rubrobacter* sp.) were the top 5 hub scores.

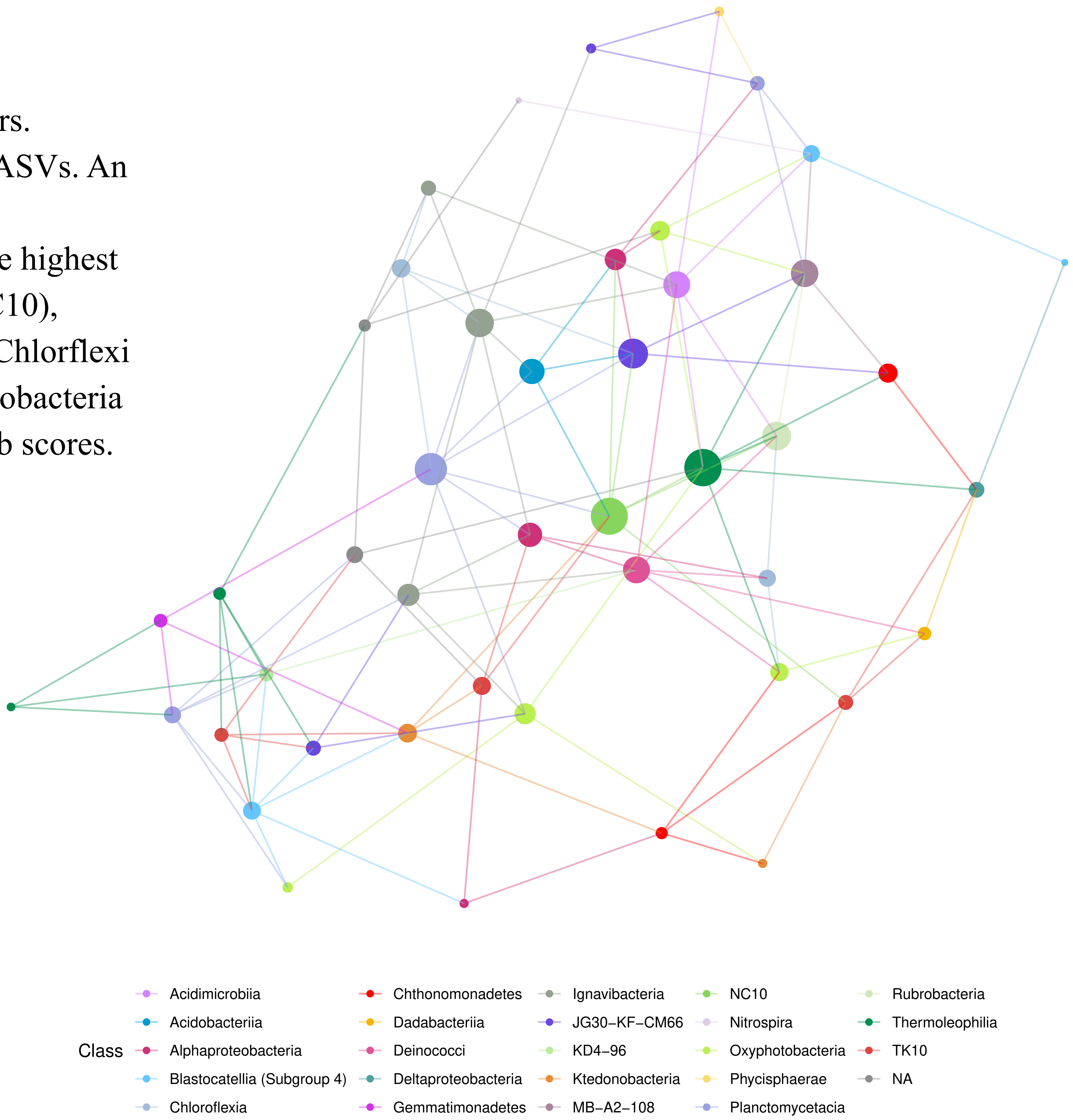

Consortia #3

Consortia # 3 consists of 17 members, consisting of 41.2 % ASVs identified as Chloroflexi. A Ktedonobacteria had the highest hub score. A Chloroflexi (class JG30-KF-CM66), Thermoleophilia (Gaiellales) Chthonomonadetes (*Chthonomonas* sp.), and an Alphaproteobacteria (Xanthobacteraceae) had the highest hub scores within this consortia.

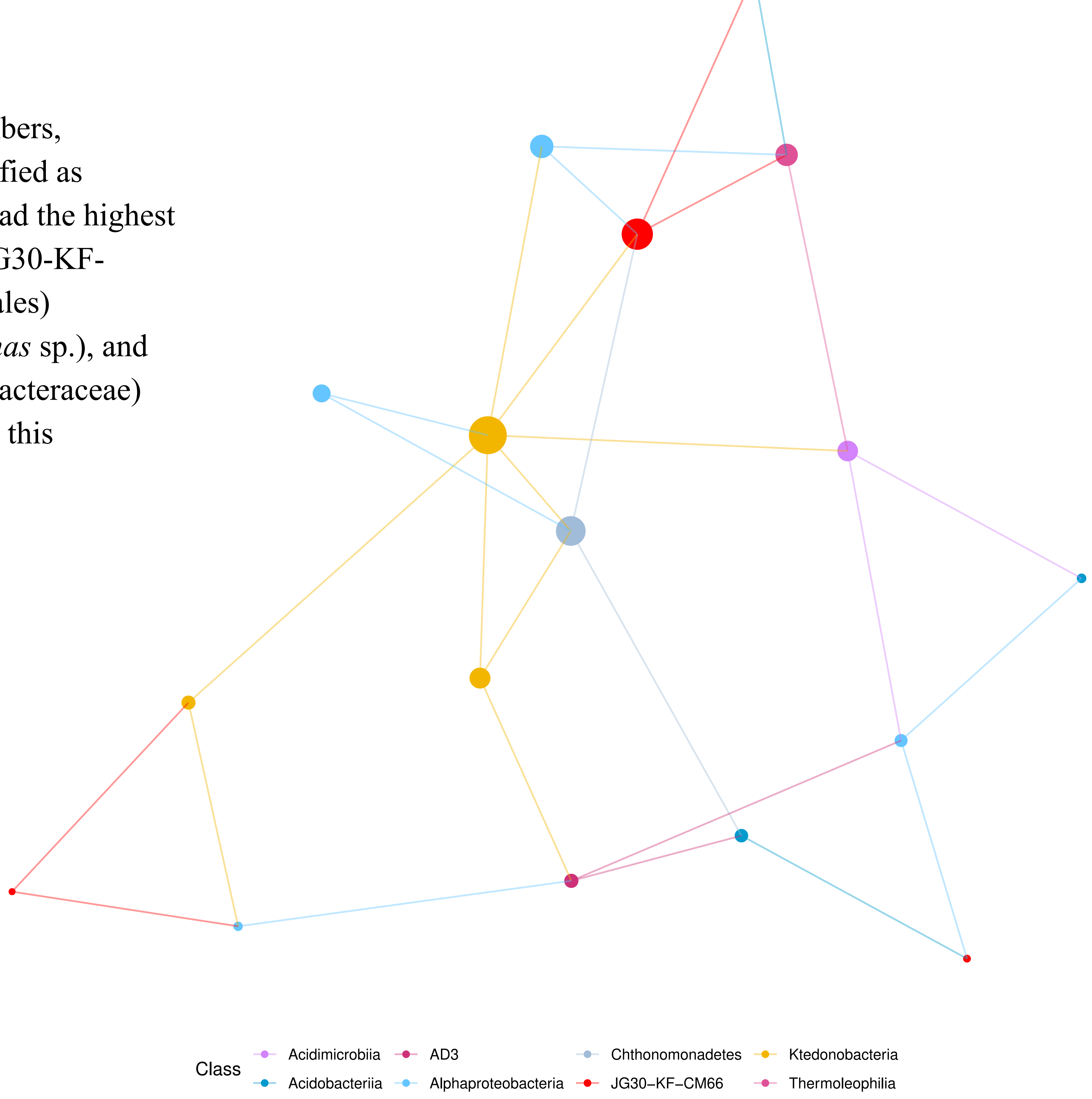

Consortia #4

Consortia # 4 consists of 53 members made of ASVs identified mostly as Proteobacteria (35.8 %) and the Cyanobacteria class Oxyphotobacteria (24.5 %). A Rhizobiales (*Hyphomicrobium* sp.) had the highest hub score. Two Solibacterales , both *Bryobacter* sp., a Oxyphotobacteria (Nostocales), and another Alphaproteobacteria (Acetobacteraceae) consisted of the top hub scores. Chloroflexi made up 11.3 % of the consortia, but none had above average hub scores.

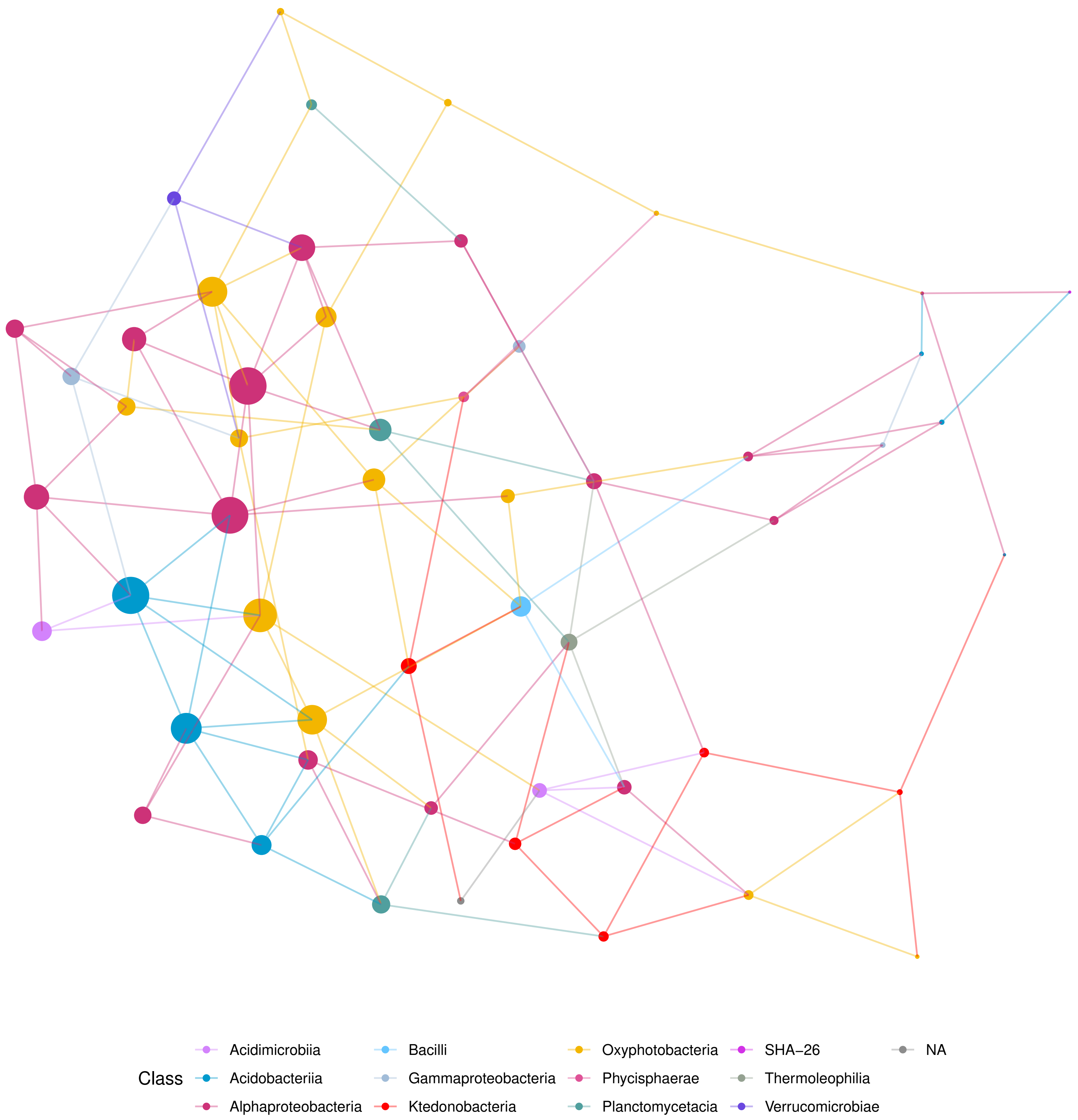

Consortia #5

Consortia #5 consists of 21 members. Proteobacteria make up 38.1 % of the ASVs, followed by Chloroflexi (28.6 %). An Alphaproteobacteria (Xanthobacteraceae) had the highest hub score. An Acidobacteriia (*Bryobacter* sp.), a Bacilli (*Bacillus* sp.), an Alphaproteobacteria (Xanthobacteraceae), and a Deltaproteobacteria (order RCP2-54) had the highest hub scores. One other ASV with a hub score within the top 5 scores was classified as an Archaea, belonging to the phylum Thaumarchaeota and class Nitrososphaeria (genus Candidatus Nitrosotalea).

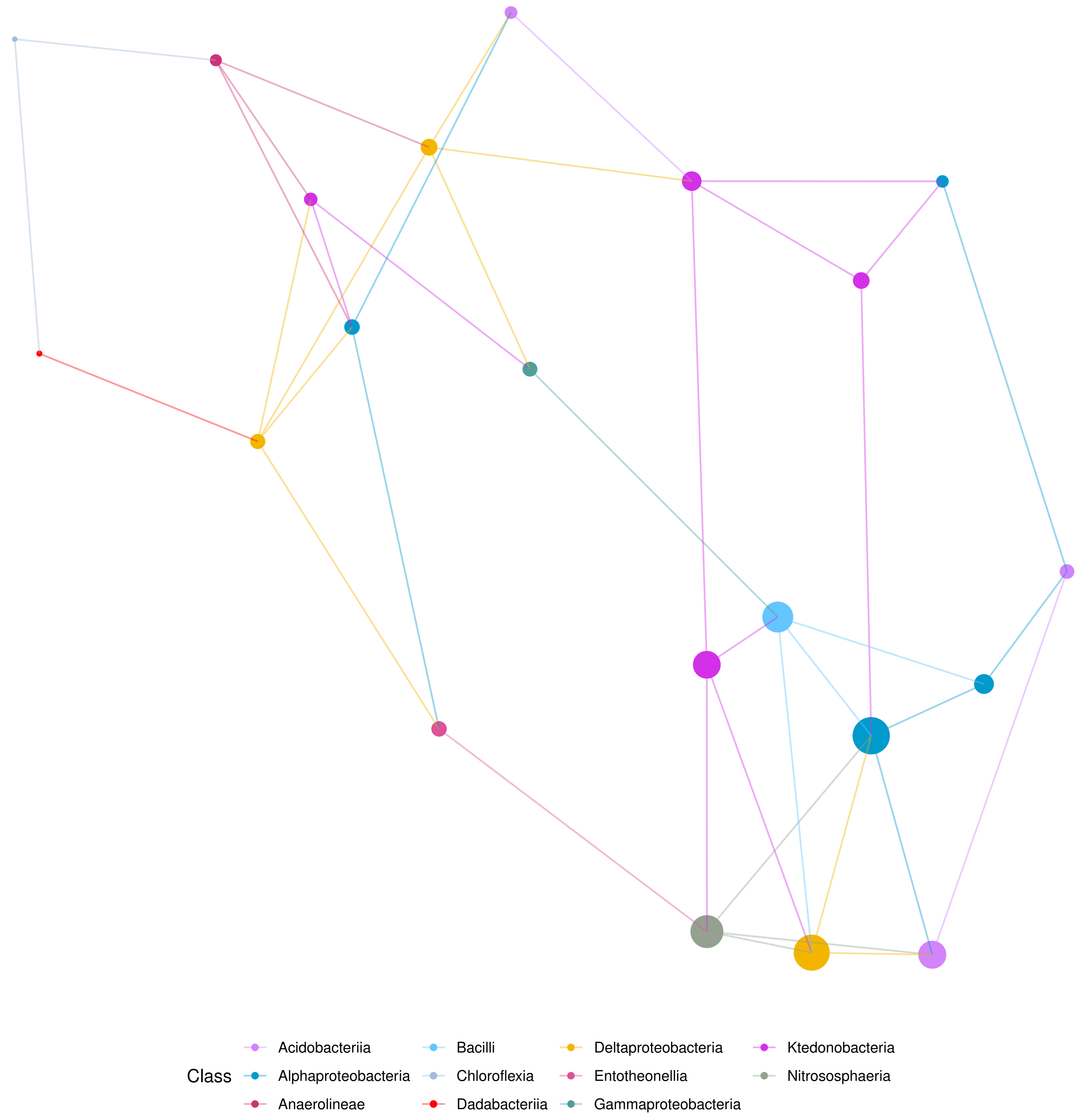

Consortia #6

Consortia # 6 consists of 14 members, with 50 % of the ASVs identified as Proteobacteria. Followed by Acidobacteria (23 %). An Alphaproteobacteria (Elsterales) had the highest hub score. An Acidimicrobiia (order IMCC26256; node 73), and three other Proteobacteria had the highest hub scores. The Proteobacteria were classified as an Alphaproteobacteria (*Reyranella* sp.; node 157), a Gammaproteobacteria (*Acidibacter* sp.; node 136, and a Gammaproteobacteria (order WD260; node 133).

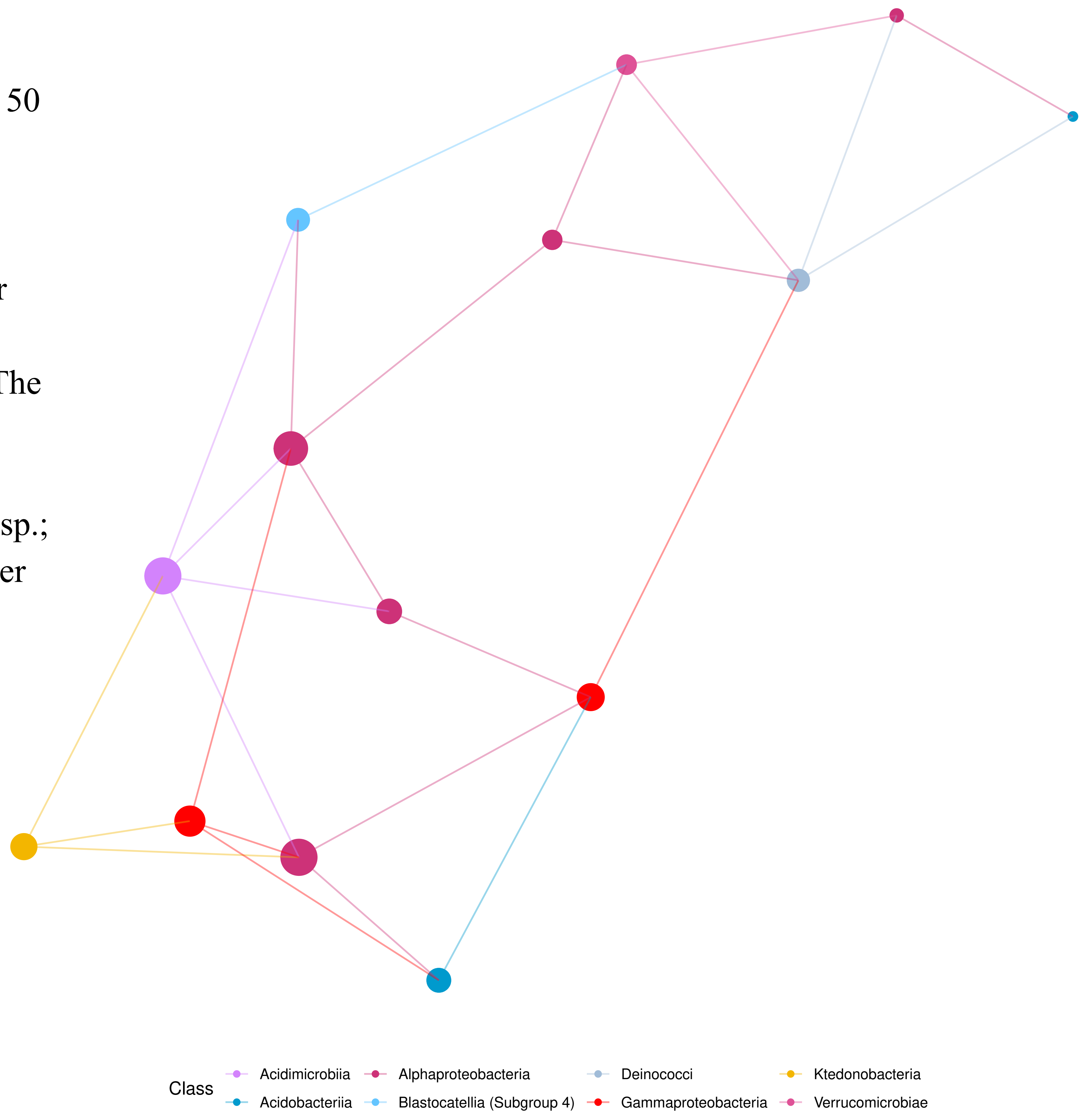

Consortia #7

Consortia # 7 consists of 39 members, with 28.2 % of the ASVs identified as Acidobacteria, mostly class Acidobacteriia. Chloroflexi and Proteobacteria both make up 20.5 % of the consortia. Acidobacteriia (Acidobacteriales) had the highest hub score. Two other Acidobacteriia, a *Bryobacter* sp. (node 200) and genus PAUC26f (node 205) also had top hub scores, as well as an ASV identified as Acidobacteriia (order IMCC26256; node 48). One Chloroflexi ASV (class JG30-KF-CM66) was also among the five top hub scores.

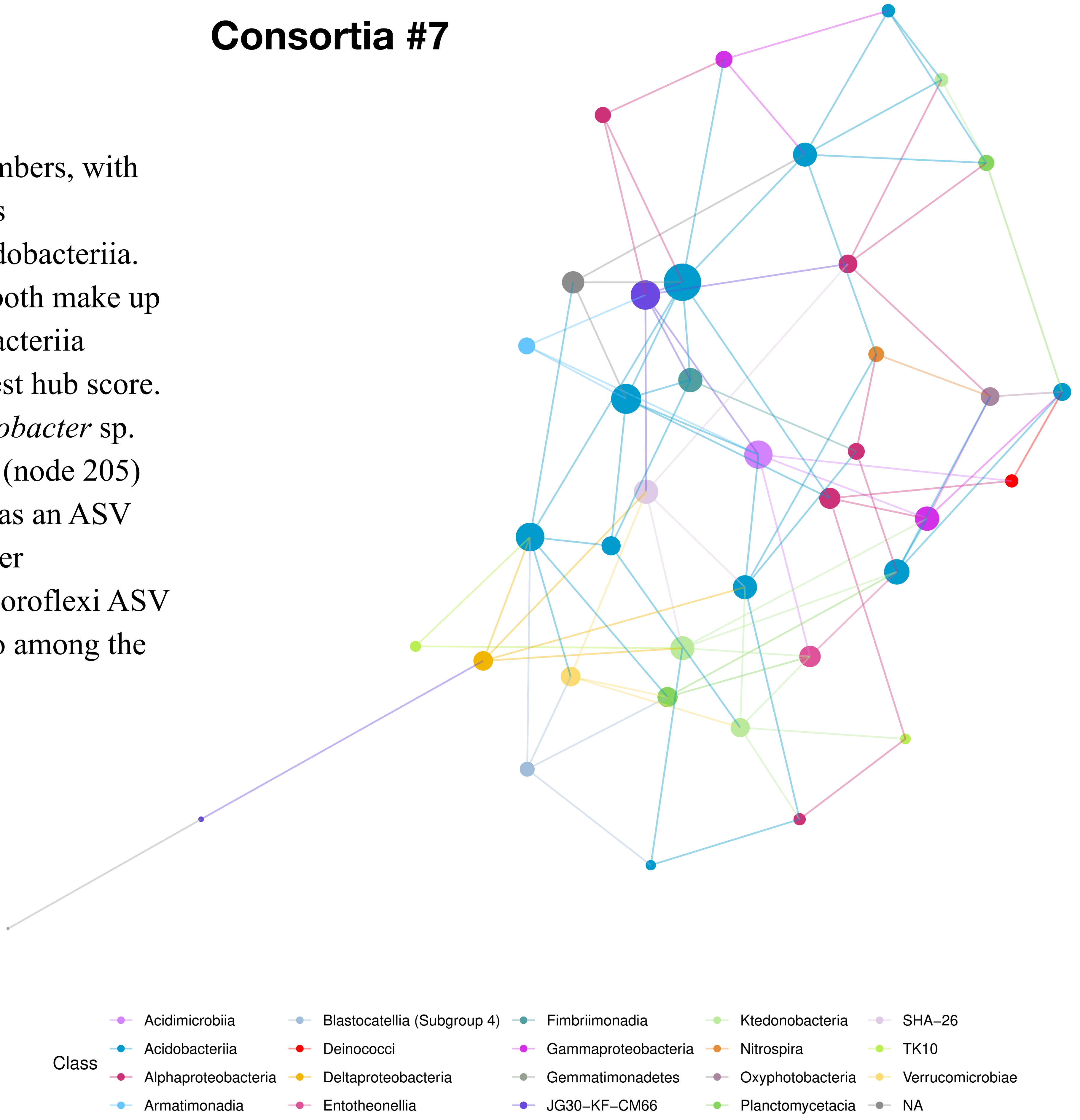

Supplement: Supplementary file 9 [file Data_Sheet_4.PDF]
