# Supplementary material for: Islands Within Islands: Bacterial Phylogenetic Structure and Consortia in Hawaiian Lava Caves and Fumaroles
Source: Front Microbiol. 2022 Jul 21;13:934708. doi: 10.3389/fmicb.2022.934708 (PMC9349362; doi:10.3389/fmicb.2022.934708)

Supplementary Figure 6: A phylogenetic tree of all ASVs in subnetworks from geothermal sites, highlighting the taxonomy of ASVs that occurred in more than one consortium (red boxes), and ASVs that had the highest hub score within each of the eight subnetworks (blue boxes). The most abundant phyla (Cyanobacteria, Chloroflexi, and Proteobacteria) are highlighted. ASVs are given to the lowest unambiguous taxonomic identification in the tree. Circles after the names of the ASVs identify the various consortia a specific ASV occurs in, with a larger circle indicating that more than one ASV is represented by that branch of the tree and occurred more than once in a given consortium (i.e., ASVs clustered at more than 98.6% similarity were grouped together within that branch), thereby suggesting that these sequences may be from the same species or strains.

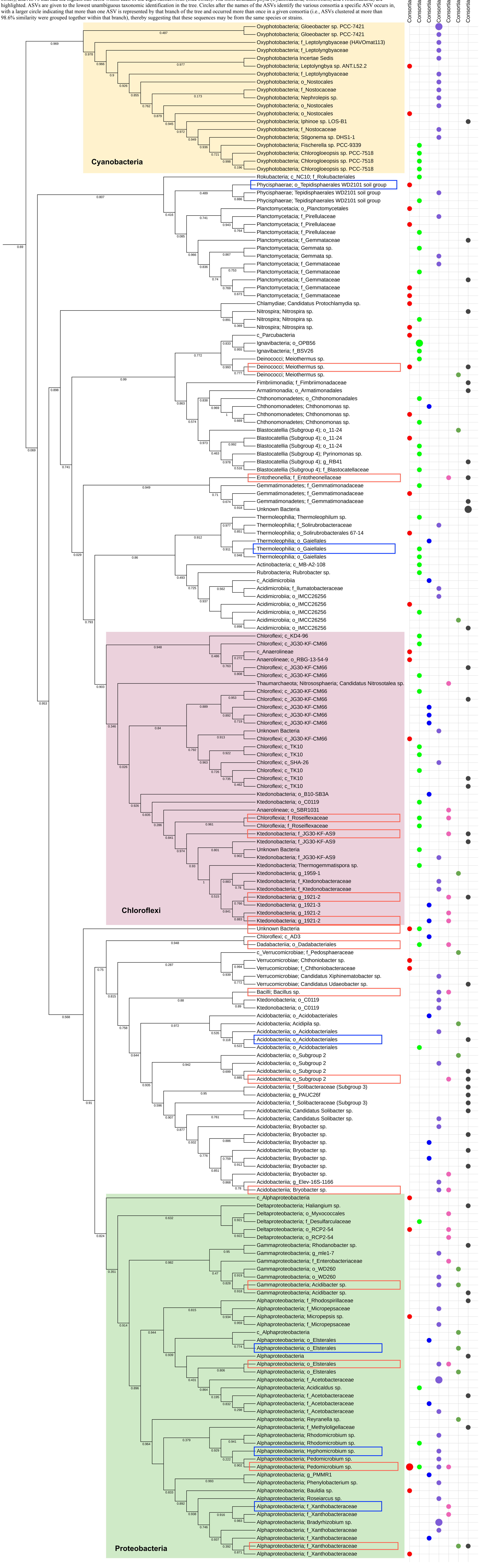

Supplement: Supplementary file 10 [file Data_Sheet_5.PDF]
