# Supplementary material for: Islands Within Islands: Bacterial Phylogenetic Structure and Consortia in Hawaiian Lava Caves and Fumaroles
Source: Front Microbiol. 2022 Jul 21;13:934708. doi: 10.3389/fmicb.2022.934708 (PMC9349362; doi:10.3389/fmicb.2022.934708)

Lava_tubes_Consortia #1

Consortia #1 has 28 members, with Actinobacteria making up 32.1 % of the consortia, followed by Proteobacteria (28.6 %). The ASV with the highest hub score was identified as Blastocatellia (Subgroup 4), in the family Pyrinomonadaceae. Other ASVs with the highest hub scores included one Ignavibacteria (order OPB56), a Longimicrobia (Longimicrobiaceae), a Planctomycetes (class OM190), and a Gammaproteobacteria (Burkholderiaceae). Chloroflexi were also present, all with above average hub scores.

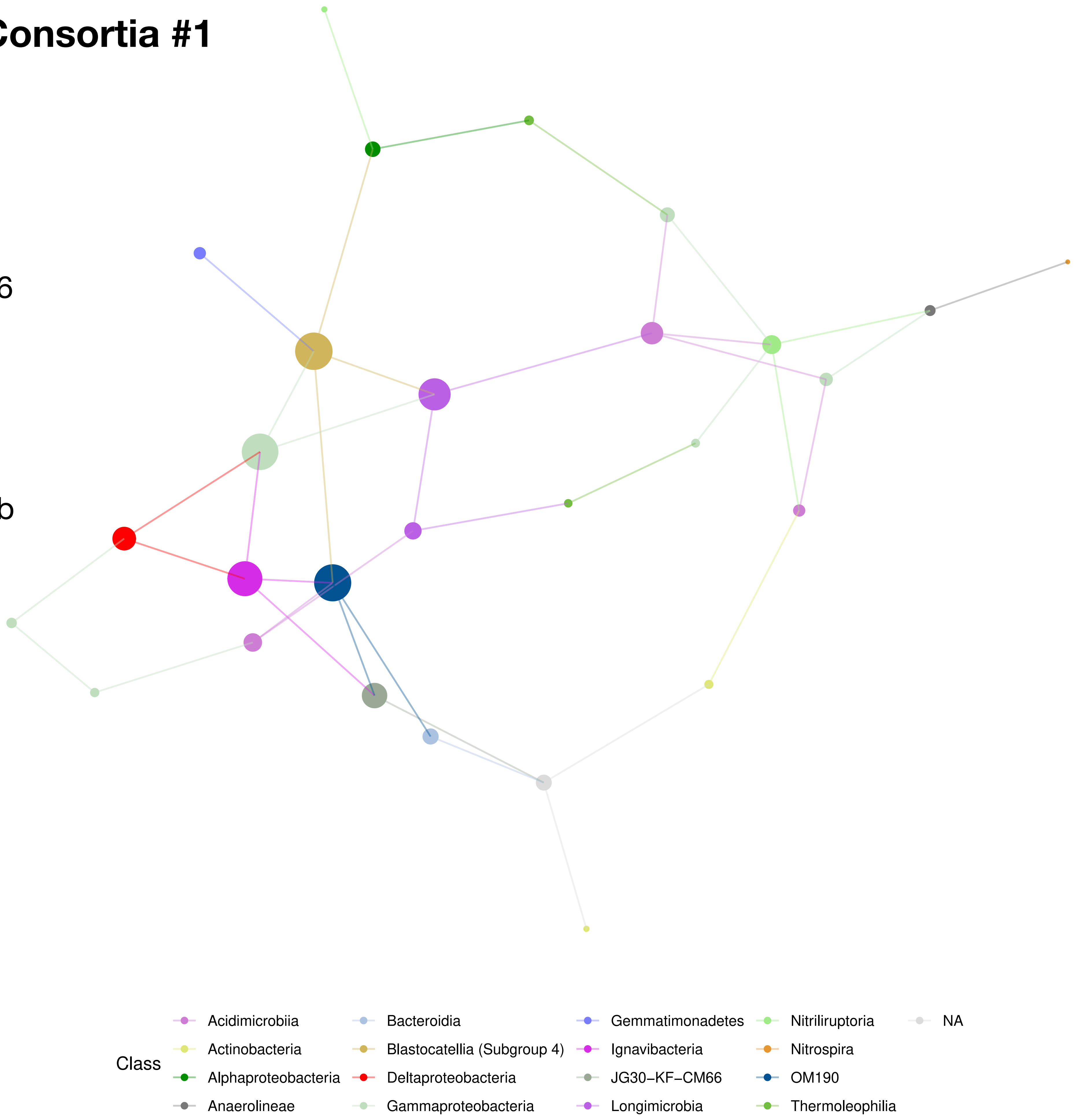

Lava_tubes_Consortia #2

Consortia #2 consists of 64 members. Proteobacteria, largely Alphaproteobacteria and Gammaproteobacteria, make up 35.9 % of the consortia. An Alphaproteobacteria of the family Hyphomicrobiaceae, had the highest hub score. Other ASVs with the highest hub scores included: Actinobacteria (class MB-A2-108), Acidobacteria (Subgroup 9), Blastocatellia (Subgroup 4) belonging to the family Blastocatellaceae, and a Deltaproteobacteria (*Haliangium* sp.).

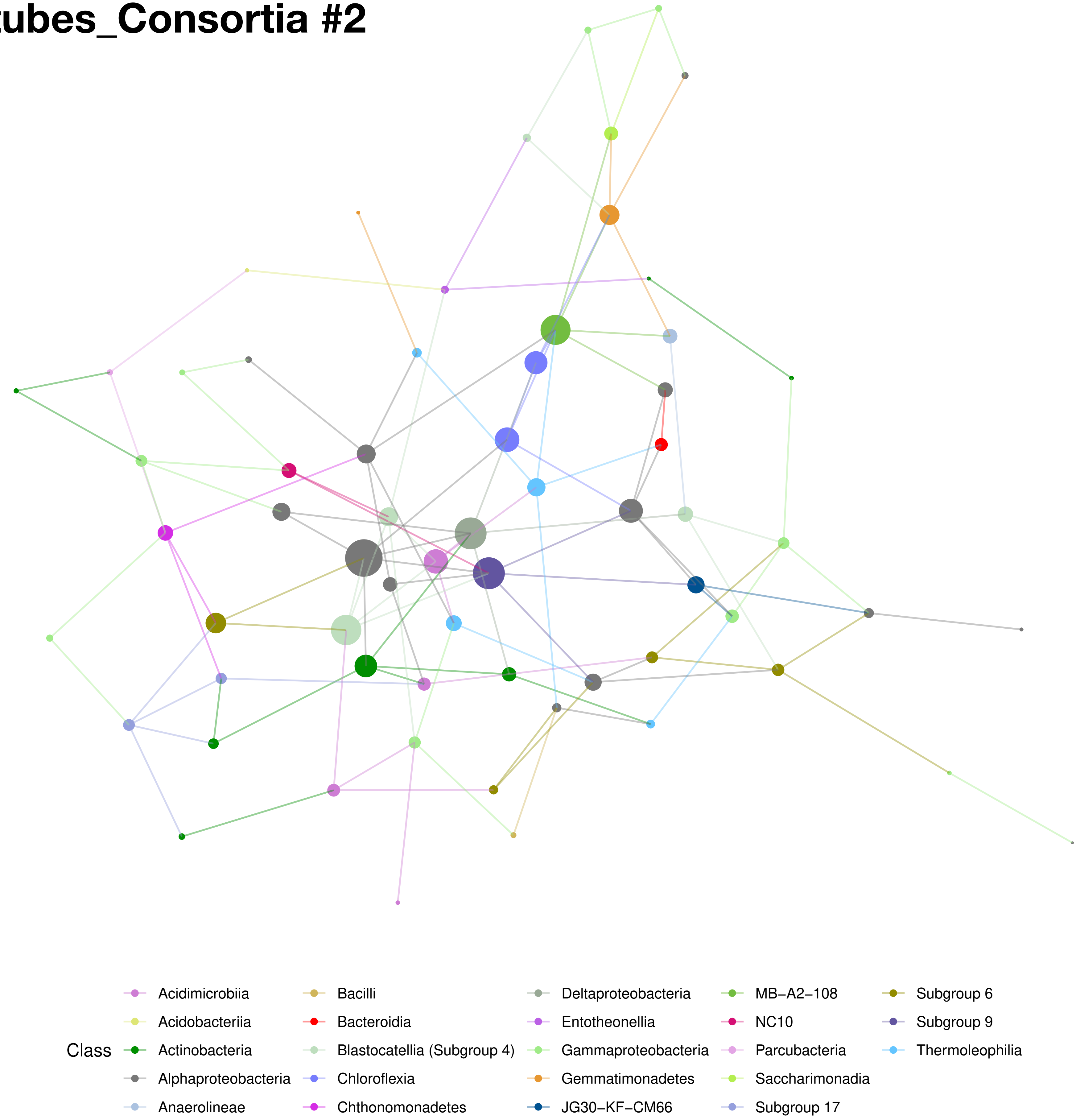

Lava_tubes_Consortia #3

Consortia #3 consists 34 members, with 41.1 % of those being Proteobacteria. A Gammaproteobacteria belonging to order Betaproteobacteriales had the highest hub score. A class Actinobacteria (*Crossiella* sp.) and two Chloroflexi, both Anaerolineae, also had the highest hub scores. Additionally, a Gammaproteobacteria (*Steroidobacter* sp.) and an Alphaproteobacteria (*Pedomicrobium* sp.) also had the highest hub scores.

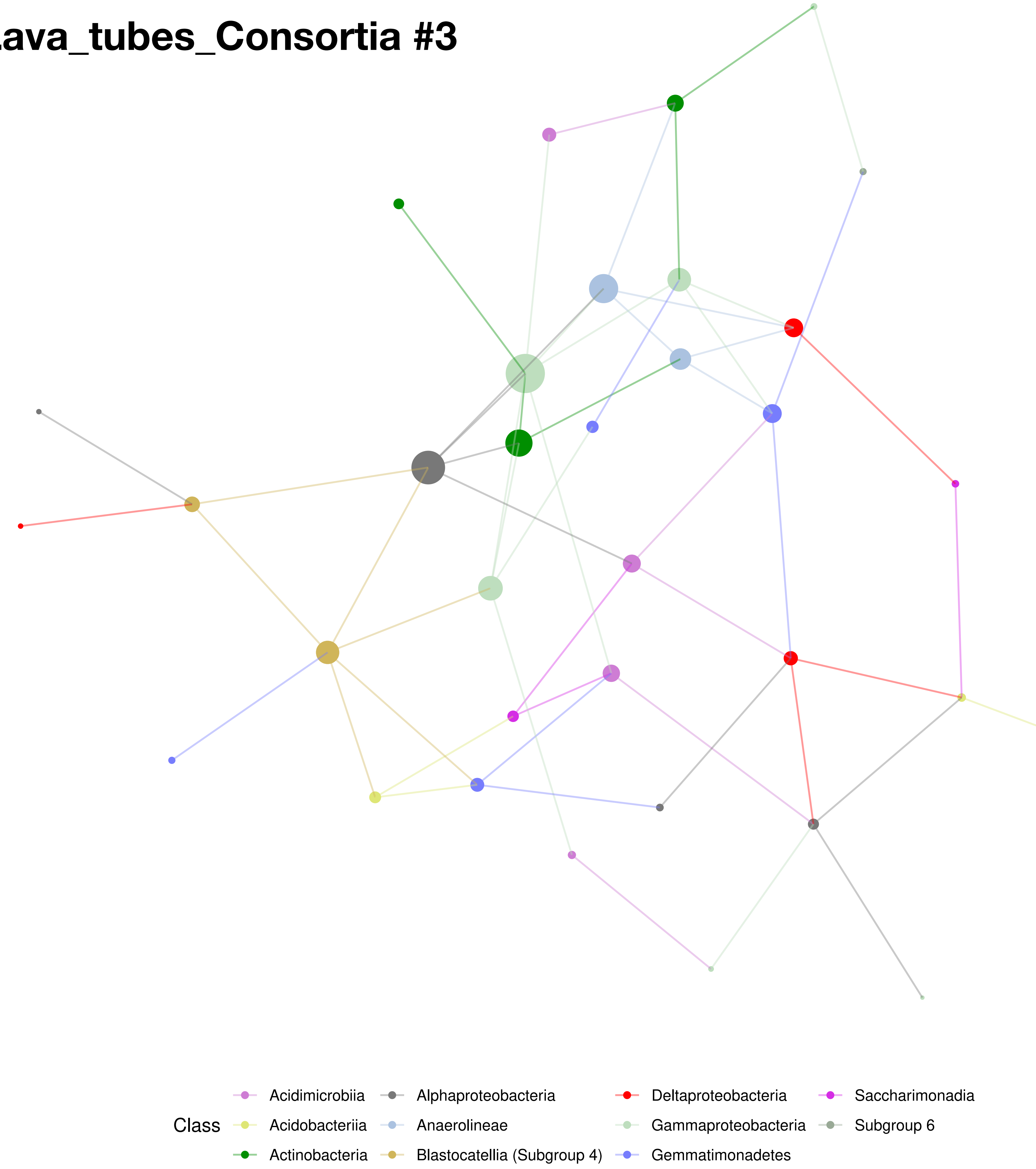

Lava_tubes_Consortia #4

Consortia #4 contains of 13 members, dominated by Actinobacteria and Proteobacteria, both 30.7 %. Chloroflexi, Gemmatimonadetes, and Planctomycetes make up the other groups present in this consortia. A Nitriliruptoria (Nitriliruptoraceae) had the highest hub score. The other top 4 hub scores included ASVs identified as class Actinobacteria (*Pseudonocardia* sp.), Anaerolineae (Caldilineaceae), Longimicrobia (Longimicrobiaceae), and Phycisphaerae (Phycisphaeraceae).

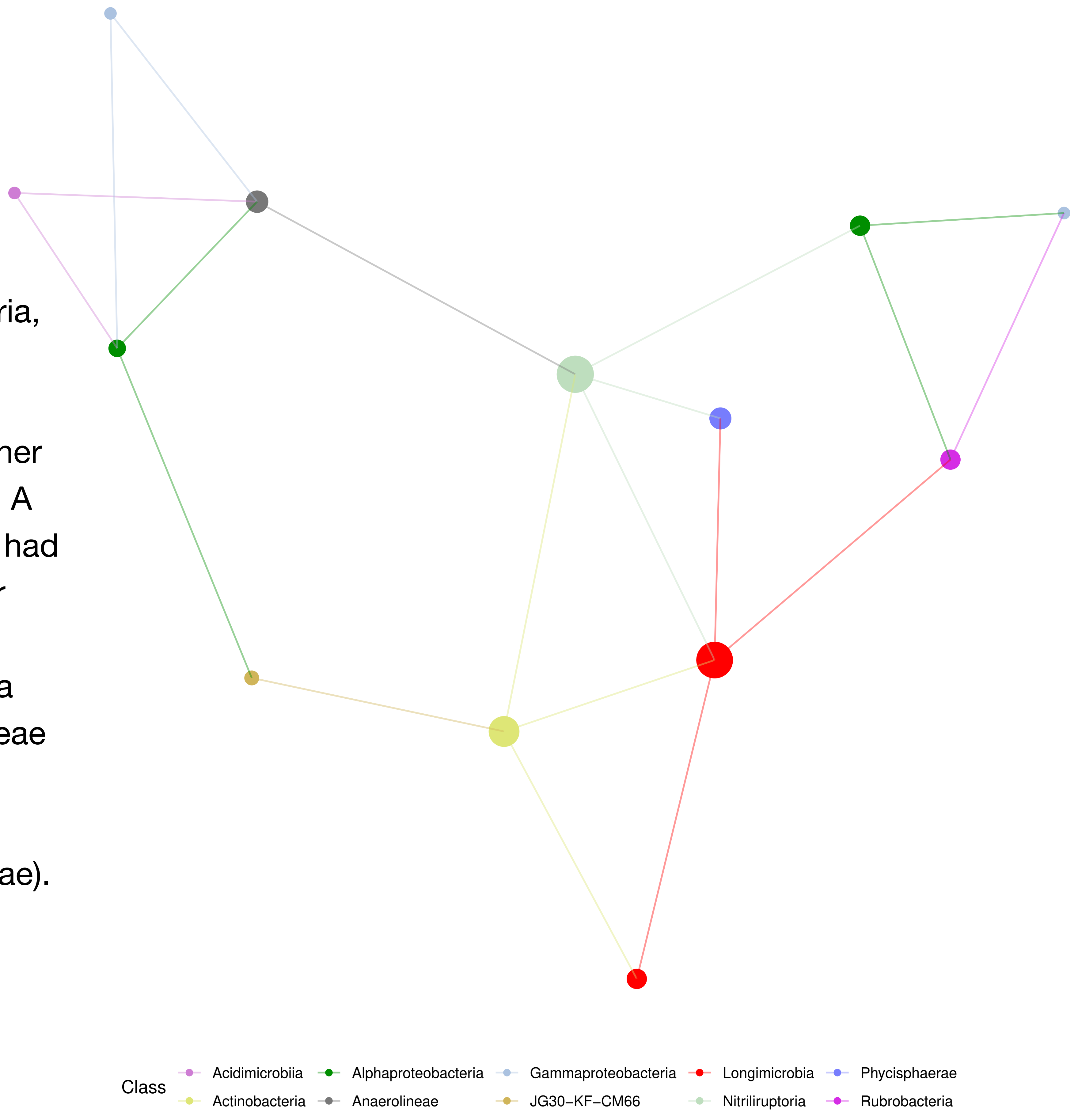

Lava_tubes_Consortia #5

Consortia #5 contains of 54 members, dominated by Proteobacteria (44.4 %). A Blastocatellia (Subgroup 4) had the highest hub score. An Acidobacteria (subgroup 22), Ignavibacteria belonging to order Kryptoniales (family BSV26), Saccharimonadia (Saccharimonadales), and a Gammaproteobacteria (order PLTA13) also had the top hub scores. This consortia had an Archaea of class Nitrososphaeria (Nitrosopumilaceae) with a hub score that was higher than average. There was also a Calditrichaeota (Calditrichaceae).

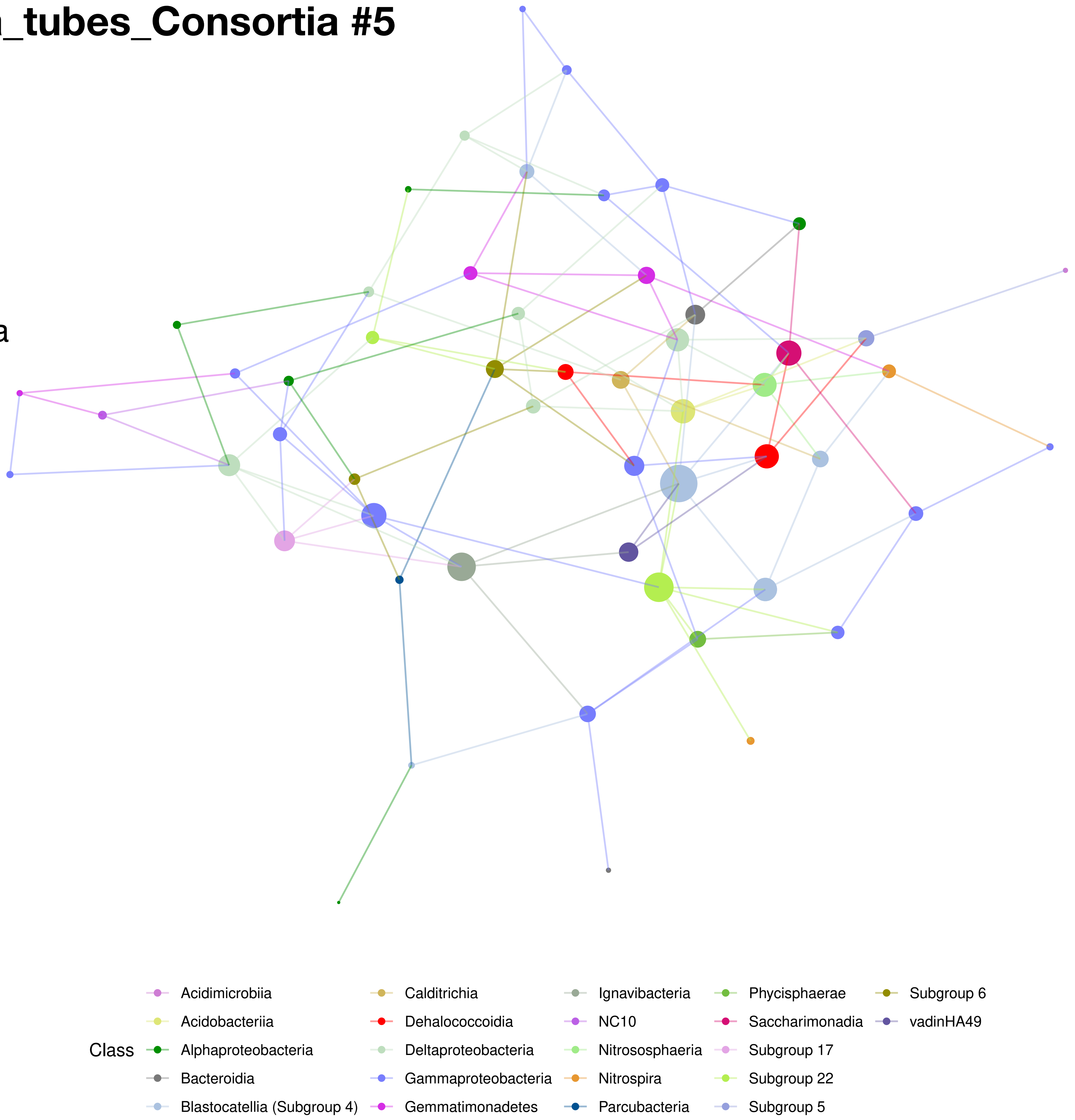

Lava_tubes_Consortia #6

Consortia #6 contains of 16 members, largely by Proteobacteria (56.3 %). An Acidimicrobiia (order IMCC26256) had the highest hub score. Other members with the top 4 highest hub scores included: Anaerolineae (order RBG-13-54-9), Nitrospira (*Nitrospira* sp.), Alphaproteobacteria (*Hyphomicrobium* sp.), and an Alphaproteobacteria of the family Methyloligellaceae. A Rukubacteria was also present, but did not have an above average hub score.

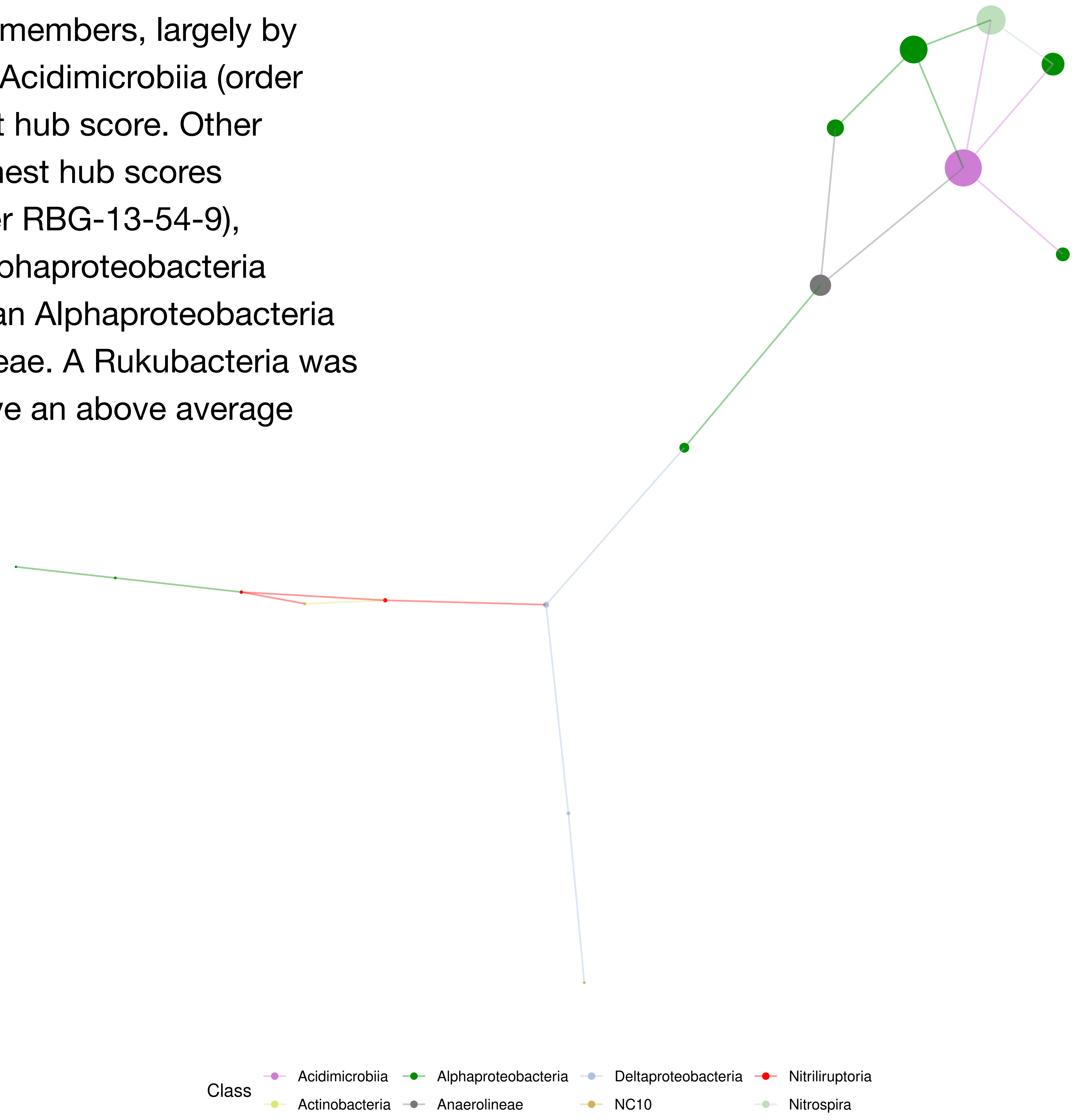

Lava_tubes_Consortia #7

Consortia #7 contains of 23 members, largely by Proteobacteria (43.5 %). An ASV identified as Gemmatimonadetes (Gemmatimonadaceae) had the highest hub score. An Acidobacteria (Subgroup 6), Deltaproteobacteria (order Myxococcales), and two Gammaproteobacteria (orders PLTA13 and Betaproteobacteriales) also had the highest hub scores. Cyanobacteria, belonging to class Melainabacteria (Obscuribacterales) were present. This was the only consortia in lava tubes that had Cyanobacteria as identified ASVs.

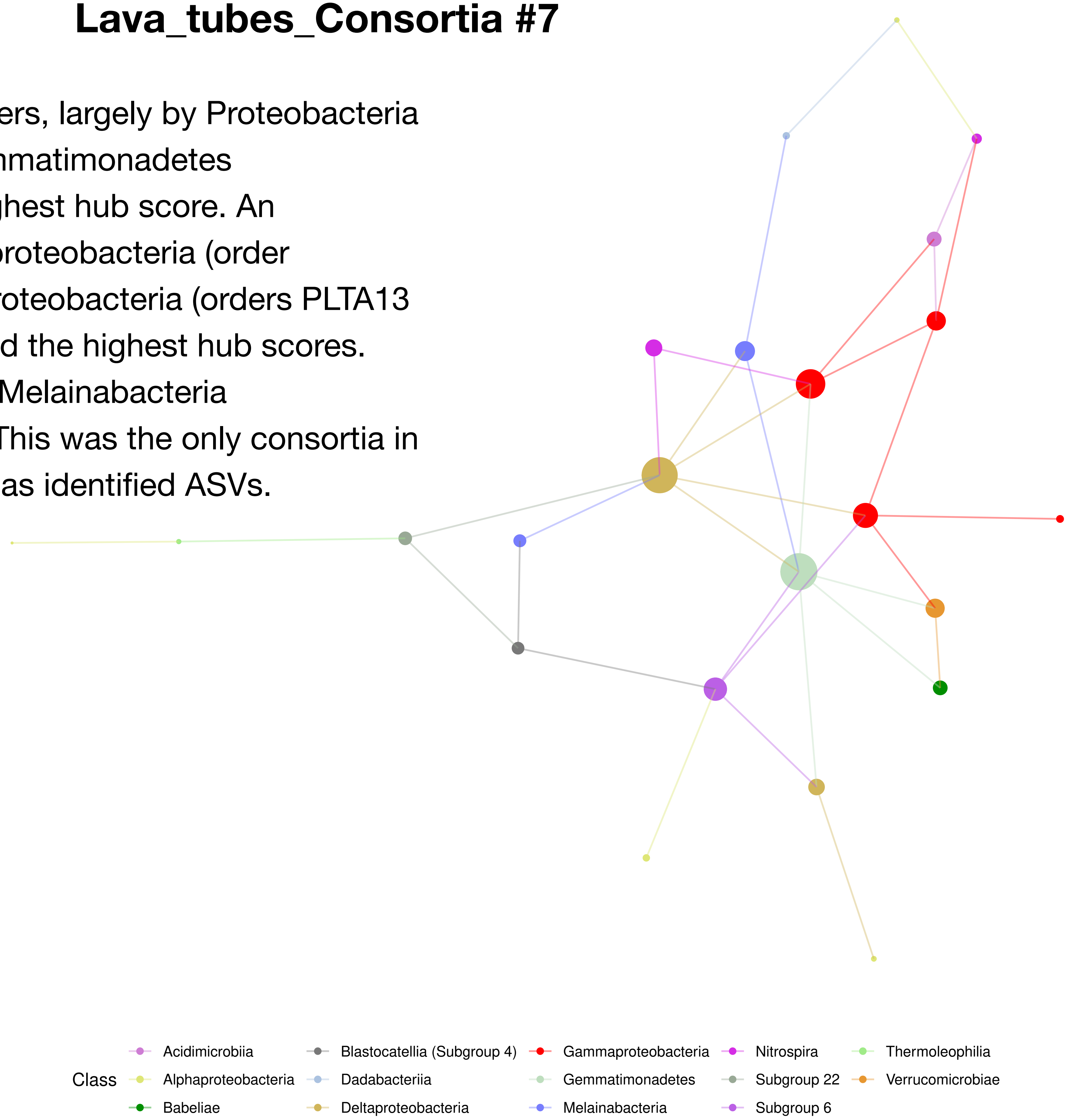

Lava_tubes_Consortia #8

Consortia # contains of 26 members. Proteobacteria make up 50% of the consortia. An ASV identified as Phylum GAL15 had the highest hub score, with a second ASV also identified as GAL15 being in the top 5 hub scores. This was the only consortia to contain ASVs identified as GAL15. Additionally, an Acidobacteria (subgroup 9), Actinobacteria (class MB-A2-108), and a Gammaproteobacteria (Nitrosomonadaceae) were also among the top 5 hub scores. As with all consortia, Chloroflexi were also members, both belonging to class Anaerolineae.

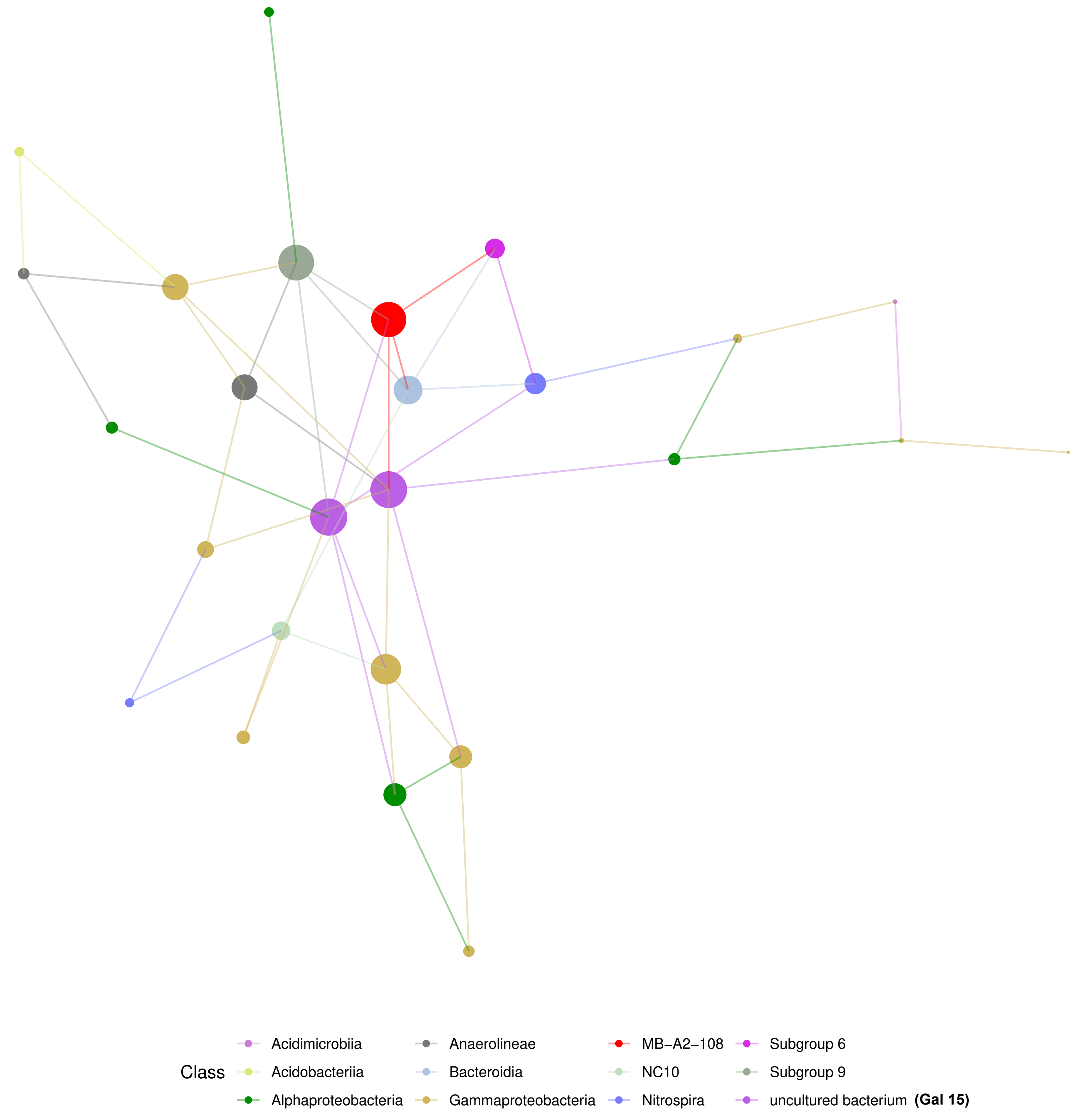

Supplement: Supplementary file 11 [file Data_Sheet_6.PDF]
