# Supplementary figures and images for: Islands Within Islands: Bacterial Phylogenetic Structure and Consortia in Hawaiian Lava Caves and Fumaroles
Source: Front Microbiol. 2022 Jul 21;13:934708. doi: 10.3389/fmicb.2022.934708 (PMC9349362; doi:10.3389/fmicb.2022.934708)

Proteobacteria

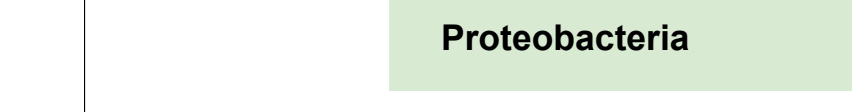

Supplement: Supplementary file 12 [file Data_Sheet_7.PDF]

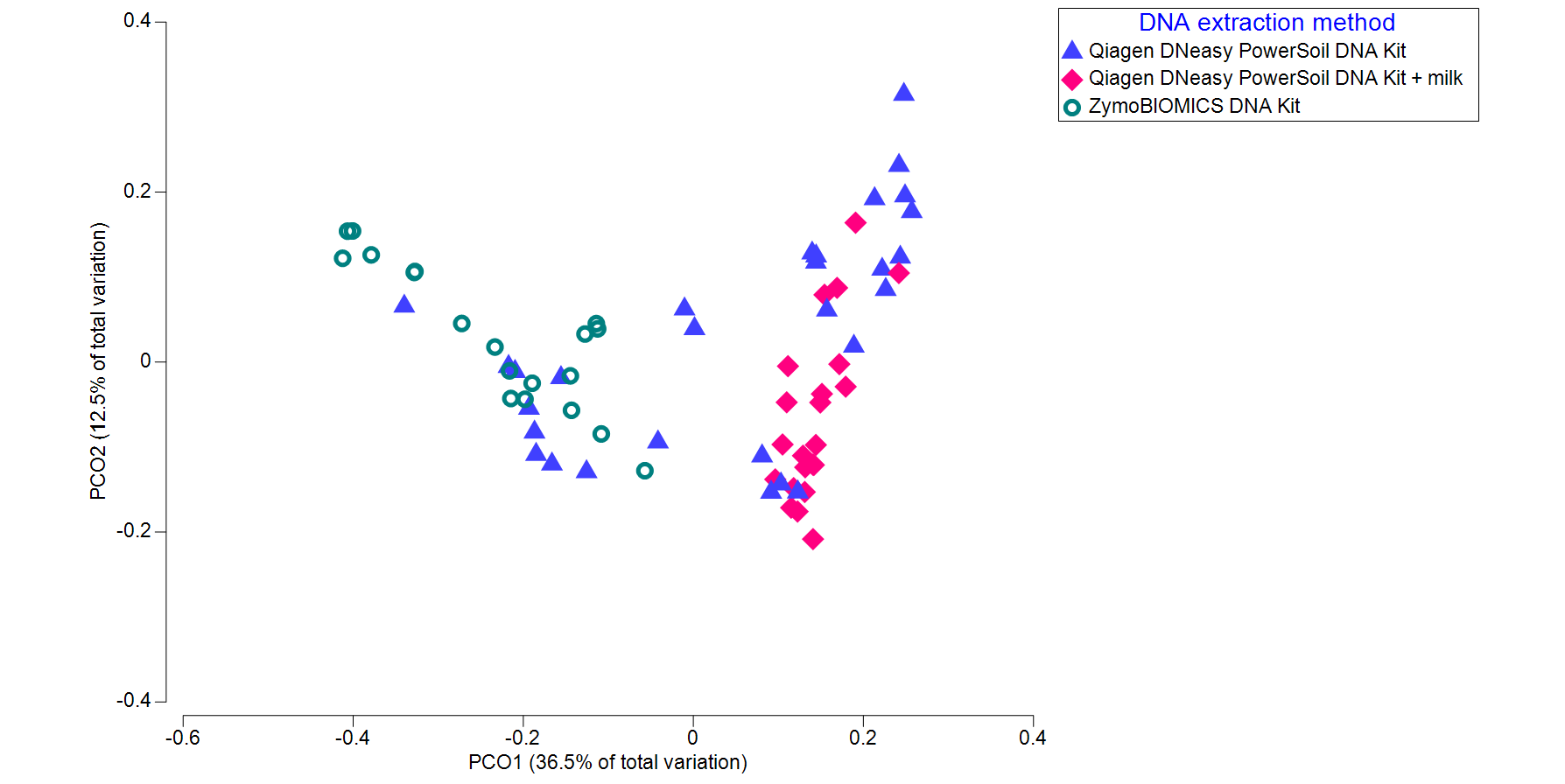

Supplement: Supplementary file 13 [file Image_1.PNG]

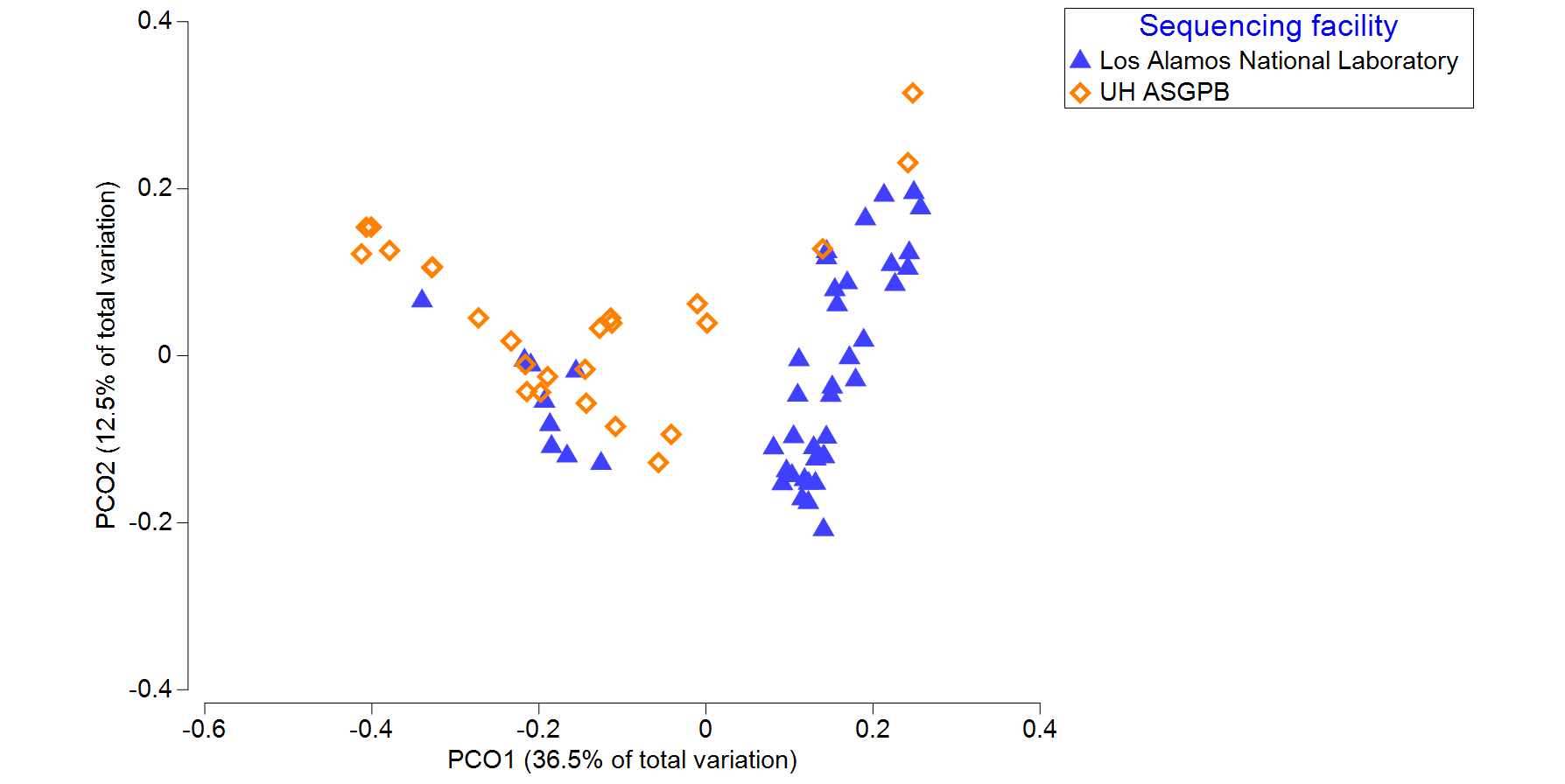

Supplement: Supplementary file 14 [file Image_2.PNG]

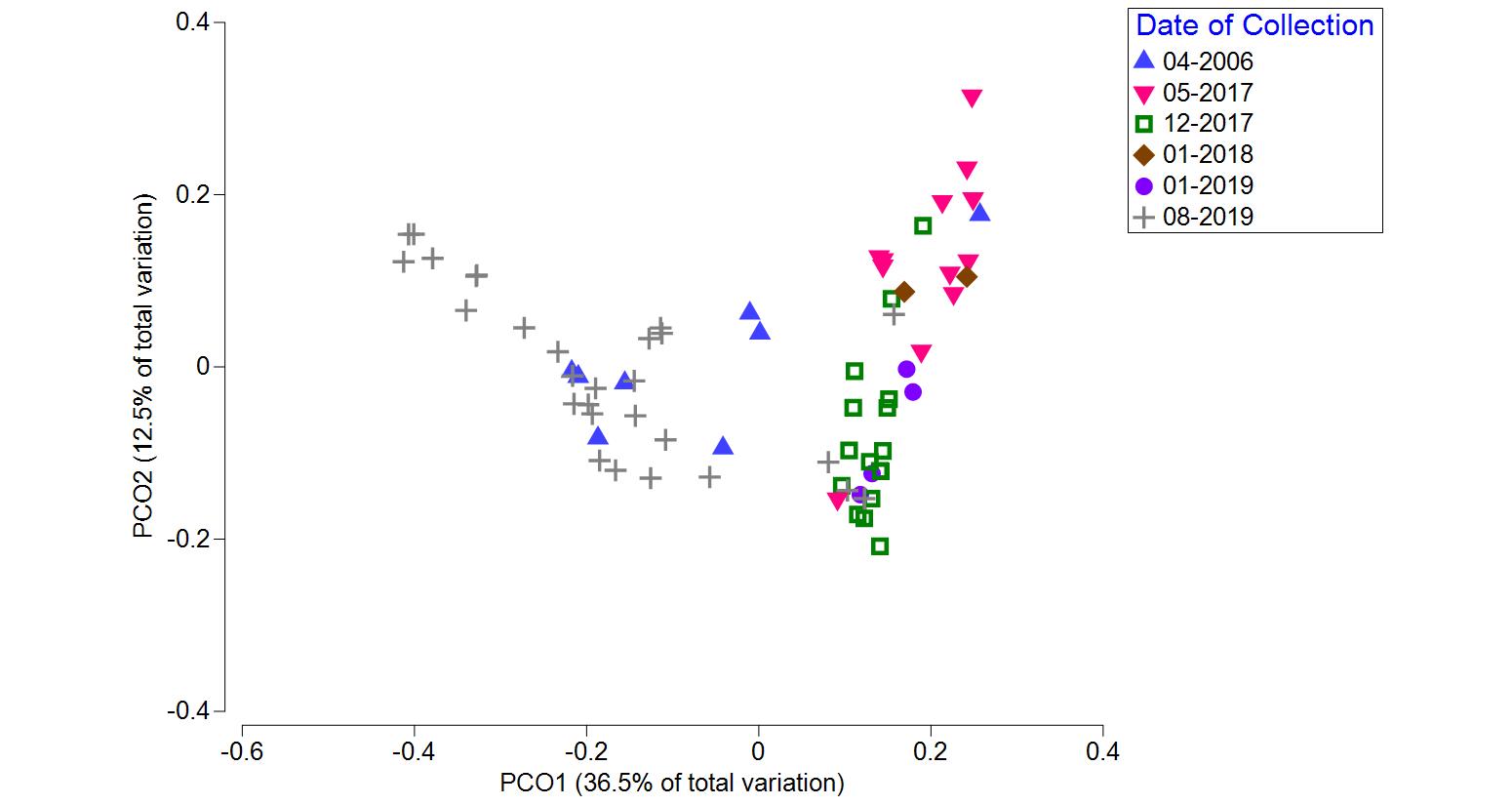

Supplement: Supplementary file 15 [file Image_3.JPEG]
